# Camel regulates development of the brain ventricular system

**DOI:** 10.1007/s00441-020-03270-1

**Published:** 2020-09-09

**Authors:** Shulan Yang, Alexander Emelyanov, May-Su You, Melvin Sin, Vladimir Korzh

**Affiliations:** 1 Institute of Molecular and Cell Biology, Agency for Science, Technology and Research, Singapore, Singapore; 2 Translational Medicine Centre, The First Affiliated Hospital, Sun Yat-sen University, Guangzhou, China; 3 Institute for Research on Cancer and Aging, Nice, France; 4 National Health Research Institutes, Zhunan, Taiwan; 5 International Institute of Molecular and Cell Biology, Warsaw, Poland

**Keywords:** Subcommissural organ, Flexural organ, Floor plate, Roof plate, Hypochord, Ependyma

## Abstract

Development of the brain ventricular system of vertebrates and the molecular mechanisms involved are not fully understood. The developmental genes expressed in the elements of the brain ventricular system such as the ependyma and circumventricular organs act as molecular determinants of cell adhesion critical for the formation of brain ventricular system. They control brain development and function, including the flow of cerebrospinal fluid. Here, we describe the novel distantly related member of the zebrafish L1-CAM family of genes—*camel*. Whereas its maternal transcripts distributed uniformly, the zygotic transcripts demonstrate clearly defined expression patterns, in particular in the axial structures: floor plate, hypochord, and roof plate. *camel* expresses in several other cell lineages with access to the brain ventricular system, including the midbrain roof plate, subcommissural organ, organum vasculosum lamina terminalis, median eminence, paraventricular organ, flexural organ, and inter-rhombomeric boundaries. This expression pattern suggests a role of Camel in neural development. Several isoforms of Camel generated by differential splicing of exons encoding the sixth fibronectin type III domain enhance cell adhesion differentially. The antisense oligomer morpholino-mediated loss-of-function of Camel affects cell adhesion and causes hydrocephalus and scoliosis manifested via the tail curled down phenotype. The subcommissural organ’s derivative—the Reissner fiber—participates in the flow of cerebrospinal fluid. The Reissner fiber fails to form upon morpholino-mediated Camel loss-of-function. The Camel mRNA–mediated gain-of-function causes the Reissner fiber misdirection. This study revealed a link between Chl1a/Camel and Reissner fiber formation, and this supports the idea that CHL1 is one of the scoliosis factors.

**Electronic supplementary material:**

The online version of this article (10.1007/s00441-020-03270-1) contains supplementary material, which is available to authorized users.

## Introduction

The subcommissural organ (SCO) is an ependymal brain gland found in the diencephalic roof of the third ventricle at the entrance to the cerebral aqueduct. It synthesizes and releases into the third ventricle (vIII) large glycoproteins that polymerize and form the Reissner fiber (RF), a filamentous structure spanning the brain ventricular system (BVS) starting from the vIII and extending through the central canal to the posterior end of the spinal cord. The RF forms by a combination of secreted proteins released to the cerebrospinal fluid (CSF) by the SCO (Nicholls 1913; Sterba 1969; Rodríguez et al. 1992, 1998; Muñoz et al. 2019). Several genes or regulators of axial midline structures including, but not limited to, the genes of the nodal and hedgehog signaling pathways (Roelink et al. 1994; Higashijima et al. 1997; Sampath et al. 1998; Lehmann and Naumann 2005) control the formation and function of cells that generate the RF such as the SCO (Sterba 1969; Oksche 1969), flexural organ (FO) (Olsson 1958), and floor plate (FP) (Rodríguez et al. 1996). The cells of the SCO (Gobron et al. 1996, 1999) and FP (Richter et al. 2001; Guinazu et al. 2002; Lehmann and Naumann 2005) synthesize and secrete the main component of the RF—SCO-spondin. RF formation takes place by polymerization of SCO-spondin and depends on additional agents (Hoyo-Becerra et al. 2005), including clusterin (Clu) and galectin-1 (lgals1) (Muñoz et al. 2019). The human SCO secretes proteins that do not polymerize and remain soluble in the CSF (Oksche 1964; Rodriguez et al. 1993, 2001). The RF disassembles at the posterior end of the central canal (filum terminale) to form the mass caudalis (Olsson 1956; Chesler and Nicholson 1985; Oksche 1969; Rodríguez et al. 1987a).

The deficiency of RF and motile cilia affects the flow of the CSF and results in hydrocephalus and scoliosis (Cifuentes et al. 1994; Grimes et al. 2016). Hydrocephalus is a common phenotype caused by developmental defects in abnormal production of CSF by the ependyma or CSF flow (Jiménez et al. 2001; Zhang et al. 2006; Kahle et al. 2016; Shen et al. 2016). Hydrocephalus correlates with mental retardation, schizophrenia, and neurodegeneration (Angeloni et al. 1999; Frints et al. 2003; Schlatter et al. 2008; Senchenko et al. 2011; Alsanie et al. 2017). The CSF flow could be affected by the lack of communication between BVS cavities, defects of motile cilia, cardiovascular abnormalities, or deficiency of the choroid plexus (CP), SCO, and RF.

During neural development and regeneration, the proteins of the immunoglobulin superfamily play essential roles in cell-cell recognition and adhesion. These include mammalian L1-CAM, N-CAM, Nr-CAM, neurofascin, and a close homolog of L1-CAM (Chl1; Maness and Schachner 2007). In zebrafish, some of these genes exist as two copies due to partial genome duplication in teleosts, for example L1-CAM (*l1cama* and *l1camb*), neurofascin (*nfasca* and *nfascb*), and Chl1 (*chl1a*/*camel* and *chl1b*) (GRCz10, release 91). In embryonic zebrafish, the duplicated genes encoding L1-CAM (*l1cam*a and *l1cam*b) express different differentiating neurons. The functional analysis showed the role of these genes during regeneration of the CNS and memory consolidation (Tongiorgi et al. 1995; Pradel et al. 2000; Becker et al. 2004). The levels of L1-CAM and N-CAM increased in the CSF of schizophrenic patients (Poltorak et al. 1997). The L1-CAM mutations in humans cause the expansion of brain ventricles, hydrocephalus, mental retardation, and deficiency of corpus callosum (reviewed in Wong et al. 1995). In mammals, Chl1 acts in axonogenesis, whereas its deficiency causes mental retardation, schizophrenia, and neurodegeneration (Angeloni et al. 1999; Frints et al. 2003; Schlatter et al. 2008; Senchenko et al. 2011; Alsanie et al. 2017). Chl1 has been suggested as a susceptibility gene of adolescent idiopathic scoliosis in humans (Sharma et al. 2011), although other studies failed to support this link (Qiu et al. 2014).

Here, we report the developmental analysis of *camel*/‘*chl1a*’ (https://www.ncbi.nlm.nih.gov/nuccore/EU560427) in zebrafish. Given its strong expression in the circumventricular organ (CVO) and axial structures, *camel* is a useful marker of the BVS development. Its maternal transcripts distribute uniformly, unlike the zygotic transcripts that demonstrate clearly defined expression pattern, in particular in the axial structures such as FP, hypochord (HC), and roof plate (RP), as well as several CVOs such as SCO and FO. Several splice variants of camel messenger RNA (mRNA) differentially enhance cell adhesion. The morpholino (MO)-mediated knockdown of Camel affects cell adhesion and RF formation and cause hydrocephalus later on. Thus, Camel emerges as the potential regulator of cell adhesion linked to the morphogenesis of the RF and formation of the BVS.

## Materials and methods

### Animals

Zebrafish embryos were obtained from the zebrafish colony maintained according to IACUC rules at the Fish Facility of the Institute of Molecular and Cell Biology, Singapore. The stages of development were presented as hours post fertilization (hpf) at 28.5 °C (Kimmel et al. 1995). All animal experiments were carried according to the regulations of the Institutional Animal Care and Use Committee (Biological Resource Center of Biopolis, license no. 120787), which approved this study. The zebrafish AB line was used as wild-type controls. The dominant-negative Notch mutant *mib*^*ta52b*^ was described (Itoh et al. 2003). The transgenic line sqet33mi2AEt (simplified as Tg(ET33-mi2a) (http://zfin.org/ZDB-ALT-110622-3) resulting from the large-scale enhancer-trap screen carries transposon insertion in the intron of *prom1a* (ENSDARG00000039966) (Kondrychyn et al. 2009). The GFP expression in this line maps to the RP and SCO. The transgenic enhancer-trap line sqet27Et (simplified here as ET27) (Parinov et al. 2004; http://zfin.org/ZDB-ALT-110622-3) carries the transposon in the intron of *pard3* (ENSDARG00000110804.1).

### Cloning and sequencing

Complementary DNA (cDNA) m11 has been isolated fortuitously during isolation of zebrafish frizzled cDNAs (Emelyanov, unpublished). Zg2 and Zg8 corresponding to fragments of cDNA of frizzleds were used as probes to screen at low stringency the high-density arrayed cDNA library of zebrafish (stage 26 somites) containing cDNA inserts ligated into the vector pSport1 (Gibco BRL, USA) (Dheen et al. 1999), and m11 clone was subjected to the whole-mount in situ hybridization. The differentially transcribed isoforms of *camel* were synthesized as cDNA by RT-PCR (Suppl. Fig. 1 and 2) and cloned into the pCS2 vector for mRNA in vitro transcription using the mMessage mMachine T7 Transcription Kit (Thermo Fisher). Sequence analysis and compilations were performed using Gene Works and MacVector (Oxford Molecular Group, UK), SnapGene, and ClustalW software. The primers and cDNA sequences for mRNA isoforms are provided in the Supplementary Materials (primers and mRNA isoforms).

**Fig. 1:**
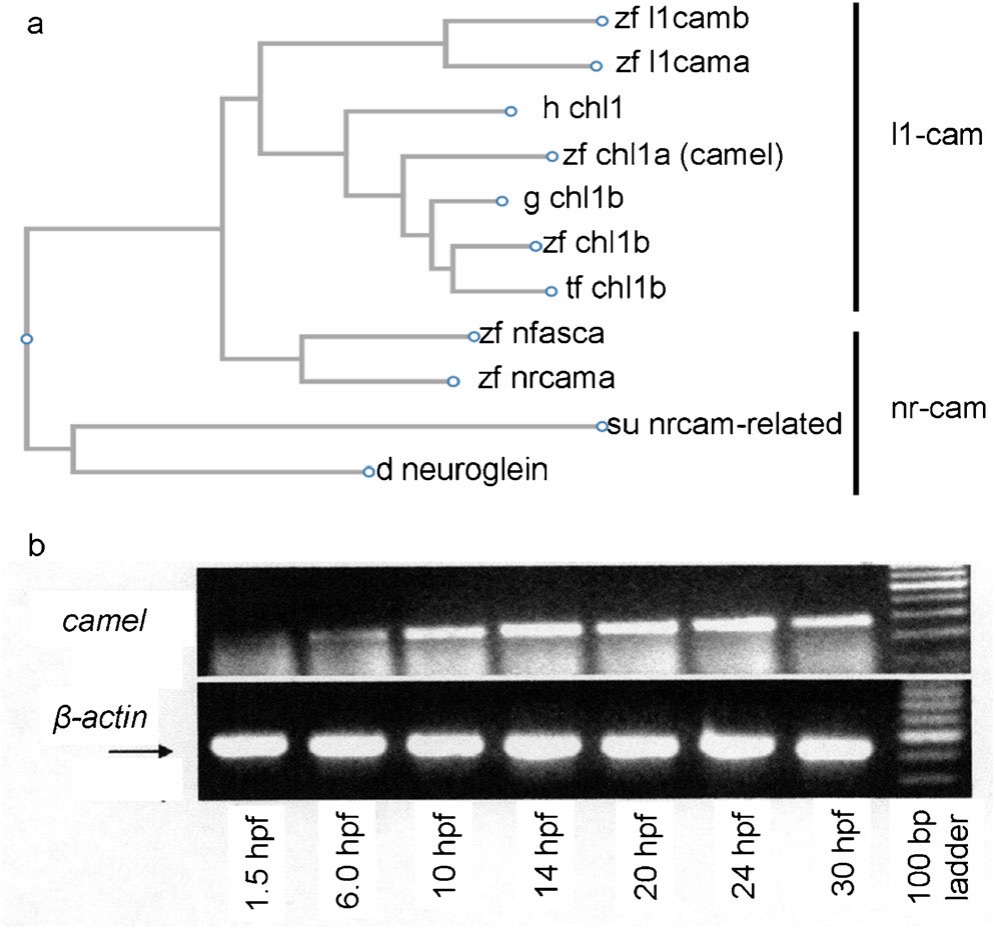
The comparison of Camel to other proteins of L1-CAM family of proteins and *camel* expression during development. Camel is the more evolutionarily distant of the two duplicated Chl1-related proteins of zebrafish. **a** The dendrogram comparison of similarity within a group of vertebrate CAM, where the Camel protein forms a distinct group different from the “Chl1b” one. **b** The temporal expression of *camel* during development as detected by RT-PCR. Abbreviations: d, *Drosophila melanogaster*; g, spotted gar (*Lepisosteus oculatus*); h, human (*Homo sapiens*); su, sea urchin (*Strongylocentrotus purpuratus*); tf, *Takifugu rubripes*; zf, zebrafish (*Danio rerio*)

**Fig. 2:**
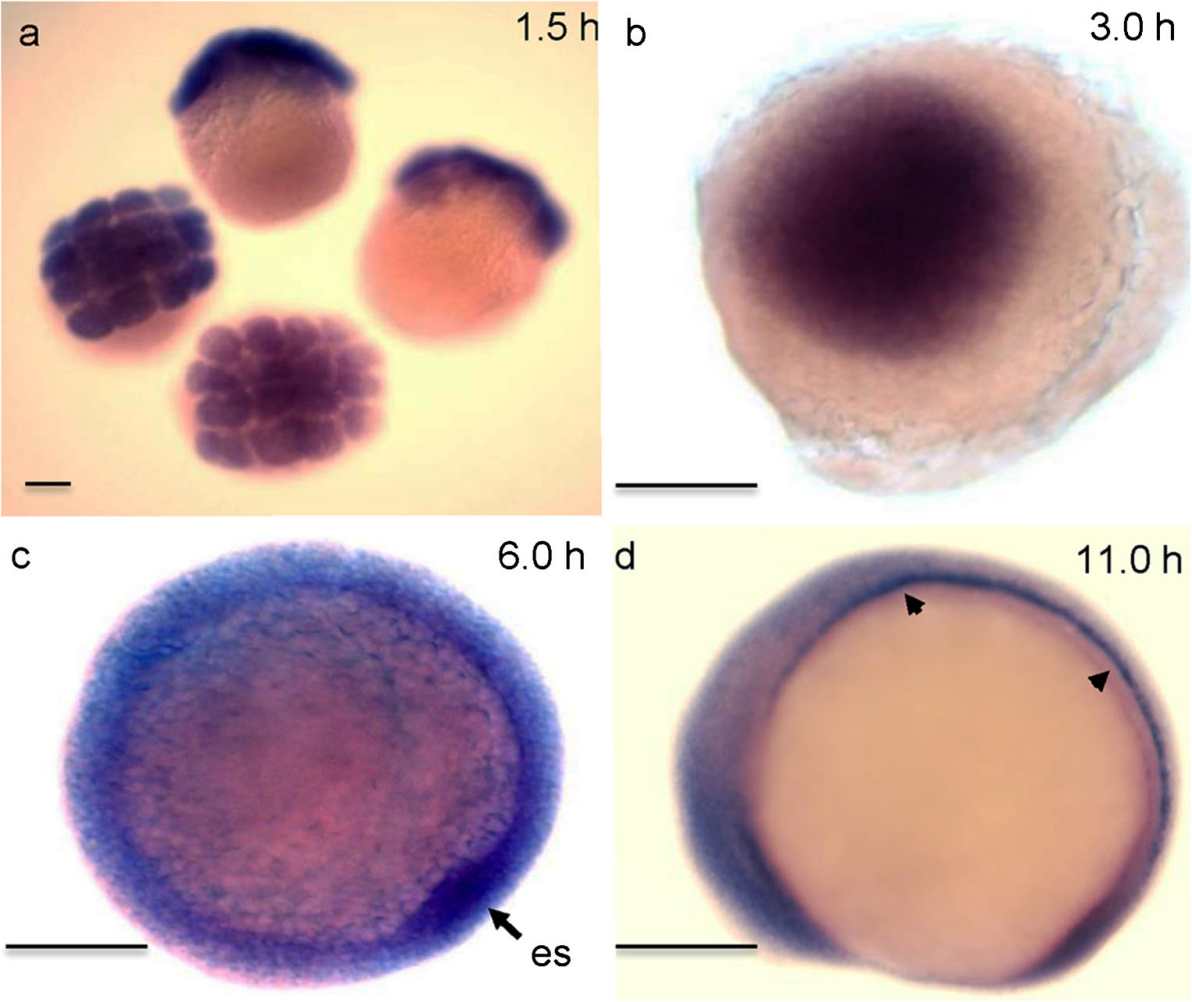
The expression pattern of *camel* during early zebrafish development detected by whole-mount in situ hybridization. **a**, **b** *camel* is inherited as a maternal transcript distributed uniformly. **c** During gastrula, the embryonic shield (ES, arrow) is stained more intensely compared to other regions. **d** During somitogenesis, the expression is largely in the ventral midline (arrowhead). Scale bar = 100 μm

### Morpholino and mRNA microinjection

All morpholino (0.2 pmol) and mRNA (100 pg) were injected at the 1–2 cell stage.

- MO1: 5′-CACTgAgACTCCTgAGCCCCCTCAT-3′ (targets ATG site)
- MO2: 5′-gAgCTTCTTACCgACTCCTTCAAgA-3′ (targets 5′-UTR, nt 55–79)
- MS1: 5′-AgCACgACTgAgAgAAATACAAAgA-3′
- MS2: 5′-ATCACCTggAggAATAACCgCATAT-3′

mRNA was synthesized from the linearized expression vector pCS2 using T7 mMessage mMachine (Amgen) in vitro transcription kit and aliquoted to be kept at − 80 °C. For co-injection, the MO and mRNA were mixed prior to injection.

Once formed, the brain fourth ventricle (vIV) was injected for visualization of the BVS with 5 kDa Texas Red at 20–22 hpf as described in Shen et al. (2016).

### RT-PCR

Total RNA from embryos at selected stages was isolated using RNeasy Kit (Qiagen, Germany); 0.5 μg total DNA-free RNA was used for multiplex RT-PCR using One-Step RT-PCR Kit (Qiagen). The primers used FP 5′-TgCAgCATTCgTgCTCAACgT-3′ and RP 5′-AACAgCCgATgAggACAAgCA-3′. The wild-type PCR product was 368 bp. The zebrafish β-actin gene was used as a control. The primers used were β-actin, forward 5′-TggCATTgCTgACCgTATgC-3′ and reverse 5′-gTCATggACgCCCATTgTgA-3′ (FP1/RP1, 450 bp). The control PCR for DNA contamination was performed without RT step. The annealing temperature for PCR was 55 °C. The aliquots of PCR mixture were removed after 25, 30, and 35 cycles to determine that the fragments were in the exponential phase of amplification. The position of primers for amplification of different regions of *camel* and its isoforms are shown in Suppl. Fig. 1 and 2.

### Whole-mount in situ hybridization and immunostaining

Whole-mount in situ hybridization (WISH) using RNA probes labeled with digoxigenin (Dig; Roche, USA) was carried out as previously reported by Oxtoby and Jowett (1993). The embryos were stained by the two-color immunohistochemistry for GFP and RF using mouse anti-GFP Mab and polyclonal rabbit AFRU antibody (1:1000) (a gift of Drs. J. Grondona [Malaga, Spain], E. Rodriguez, and M. Guerra [Valdivia, Chile]) according to the described protocol (Korzh et al. 1998).

### Microscopy

The embryos stained using WISH were prepared and observed using differential interference contrast (DIC) microscopy as described before (Korzh et al. 1998).

The temperature of the confocal microscope chamber was maintained at 28 °C during image acquisition. Imaging was performed using the microscope Zeiss LSM 800 with Airyscan (Carl Zeiss, Germany); 488-nm and 561-nm lasers were used to excite fluorescence with emission detected using emission filters (505–545 nm and 575–615 nm BP), respectively. Data were saved in the CZI format and then processed using ImageJ 1.51n software (Fiji). For each, z-stack average intensity and sum slice projections were generated.

### Hanging-drop cultures

Hanging-drop cultures were carried out with animal caps from embryos at 50% epiboly as previously described (Steinberg and Takeichi 1994; Fong et al. 2005). Fluorescent images were obtained with a Zeiss Axioplan 2 equipped with a Zeiss AxioCam HRc CCD camera (Zeiss, Germany).

## Results

### Camel cloning and sequence analysis

We fortuitously isolated a partial fragment of cDNA during a low stringency screen of the 26-somite wild-type zebrafish high-density arrayed cDNA library using probes for zebrafish *frizzleds* (Emelyanov et al., unpublished). The library contained cDNA inserts ligated into the vector pSport1 (Gibco BRL, USA) (Dheen et al. 1999). BLAST analysis demonstrated that the partial cDNA clone is distantly related to the mammalian L1-Cam family of genes. Using PCR amplification, we generated the full-sized cDNA of this gene (GenBank acc. no. EU560427). The putative protein has 45% homology with human and mouse L1-CAM and 46% homology with human CHL1. Therefore, we named it Camel (CAM-L1-related). The BLAST against the zebrafish genome mapped the gene into the position of *chl1a* (ENSDARG00000077881). A later analysis demonstrated a presence in the genome of the second ohnolog—*chl1b*. It seems that the order of identification of these genes predetermined an extension assigned to their names—“a” and “b.” The relatively evolutionary “primitive” spotted gar genome *Lepisosteus oculatus* had no teleostean duplication (Braasch et al. 2016). It contains a single *chl1b* similar to the *chl1b* of zebrafish and much less so to the *camel*/*chl1a*. The single *chl1b* of gar and other fish species without genome duplication should be renamed as *chl1* (Gasanov et al. 2020, unpublished). In the species, where a pair of ohnologs exists, the *chl1* genes must be renamed reciprocally similar to many other ohnologs that were misnamed as suggested by the evolution-based systematic synteny analysis. Hence, *camel* should be renamed as *camel*/*chl1b*. Here, we refer to this gene as *camel*.

### Expression of *camel* during development

**Fig. 3:**
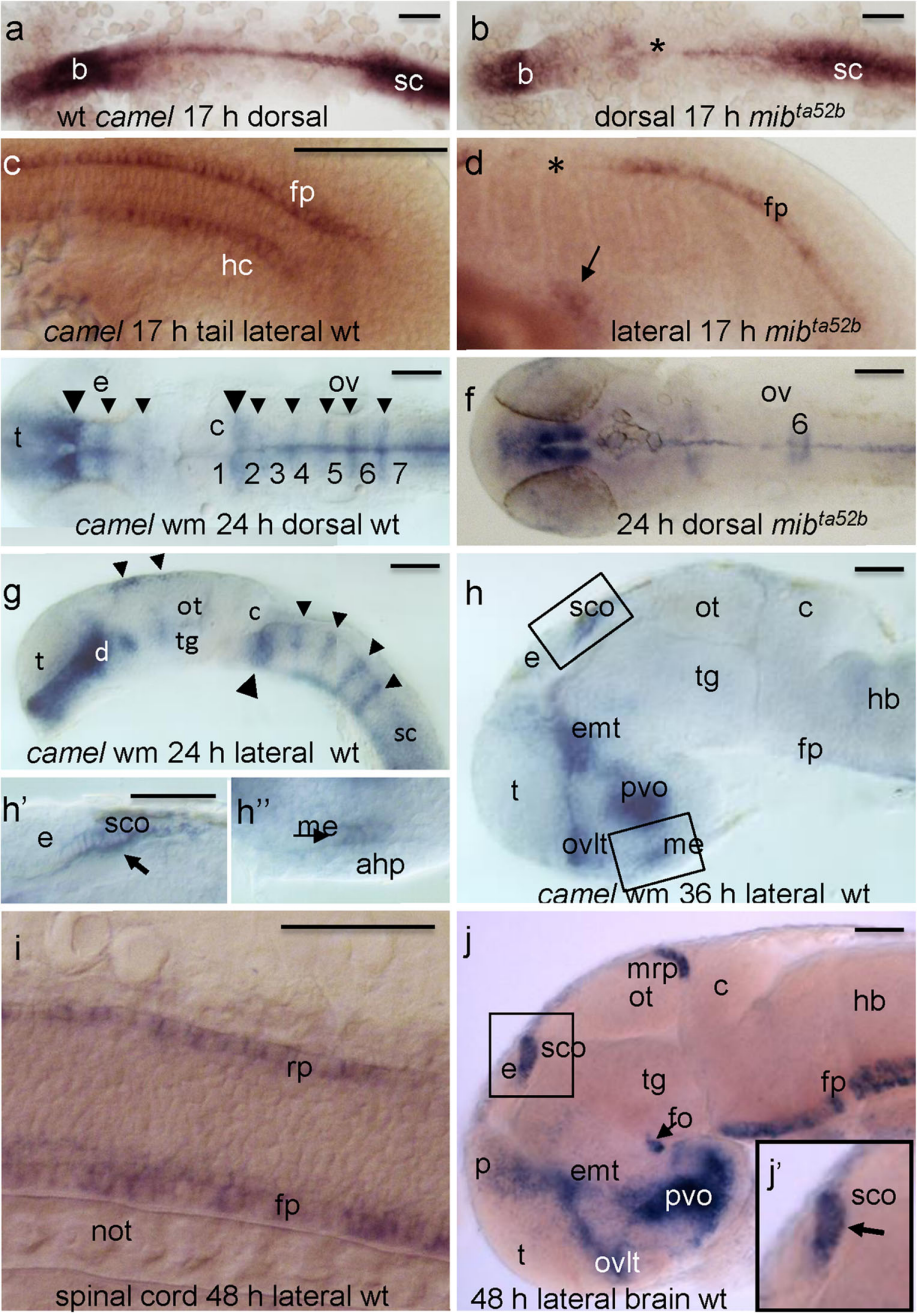
The expression pattern of *camel* in wild-type embryos and mutants detected by whole-mount in situ hybridization during neurogenesis. *camel* is expressed in the axial structures (fp, rp, hp), along segmental boundaries in the brain and in circumventricular organs. **a**, **c** 17 hpf, wild-type embryos. **b**, **d** 17 hpf, *mib*^*ta52b*^ mutant. **e**, **g** 24 hpf, wild-type embryos. **f** 24 hpf, *mib*^*ta52b*^ mutant. **h** 36 hpf, brain of a wild-type embryo. **i** Wild-type trunk, 48 hpf. **h**, **j** Wild-type brain, 48 hpf. Numbers define rhombomeres of the hindbrain, asterisk indicates gaps in the floor plate, and arrow indicates remains of hypochord. **a**, **b**, **e**, **f** Dorsal view. **c**, **d**, **g**–**j** Lateral view. Abbreviations: ahp, adenohypophysis; b, brain; c, cerebellum; d, diencephalon; e, epiphysis; emp, eminentia thalami; epIII, ependyma of the third ventricle; ey, eye; hb, hindbrain; ht, hypothalamus; fo, flexural organ; fp, floor plate; hb, hindbrain; hc, hypochord; ht, hypothalamus; me, median eminence; mrp, midbrain roof plate; not, notochord; ot, optic tectum; ov, otic vesicle; ovlt, organum vasculosum lamina terminalis; pvo, paraventricular organ; rp, roof plate; sc, spinal cord; sco, subcommissural organ; t, telencephalon; tg, tegmentum. Scale bar = 100 μm

The RT-PCR revealed the temporal expression pattern of *camel* mRNA at 1, 5, 6, 10, 14, 20, 24, and 30 hpf. It detected the presence of the maternal transcripts (Fig. 1b) and an increase in *camel* expression in a whole embryo starting from 6 hpf (Fig. 1b). The WISH revealed the spatial distribution of *camel* transcripts. Their relatively uniform distribution was detected until the beginning of somitogenesis when the anterior neural tissue and axial structures became labeled more intensely (Fig. 2a–d). In the head, at 17 hpf, *camel* was expressed more intensely in the forebrain and in the trunk (HC and FP) (Fig. 3a, c).

Interestingly, this expression pattern has changed substantially in the dominant-negative Notch mutant *mib*^*ta52b*^ characterized by the ectopic activity of delta (Itoh et al. 2003) (Fig. 3b). Here, the expression in the HC was absent, but some disorganized cells, probably representing remains of this structure, were detected close to the anus (Fig. 3d, arrow). This observation is in line with earlier results showing the role of notch signaling during HC development (Latimer and Appel 2006).

At 24 hpf, *camel* expression was found in the ventral diencephalon, hindbrain inter-rhombomeric boundaries, and midbrain RP (Fig. 3e, g). The WISH analysis of 24-hpf mutant *mib*^*ta52b*^ revealed an absence of expression at most inter-rhombomeric boundaries, excluding the boundary of rhombomeres 1–2 and borders of rhombomere 6 (Fig. 3f). This observation suggested that Camel may act in the maintenance of neural progenitors at these boundaries (Itoh et al. 2003; Wang et al. 2003). At 24–36 hpf, the most anterior portion of the midbrain RP started to change its relatively straight linear organization (Fig. 3g, h, h’). It bends and bulges ventrad (Fig. 3h’, arrow). By 48 hpf, these cells become much more elongated (Fig. 3j, j’), compared to other regions of RP.

At 36 hpf, *camel* expression associated with the BVS is obvious. Based on cell morphology and neuroanatomical landmarks and recent analysis (García-Lecea et al. 2017), the domain corresponding to the most anterior midbrain RP (posterior to epiphysis) corresponds to the SCO (Fig. 3h, h’). The expression domain immediately above the adenohypophysis corresponds to the median eminence (ME; Fig. 3h, h”). Note the characteristic linear distribution of cells in the SCO and ME (Fig. 3h’, h”) along the anterior-posterior axis reminiscent of that in the RP and FP (Fig. 3i). The expression domain at the floor of the vIII in the preoptic area most likely corresponds to the organum vasculosum lamina terminalis (OVLT). The domain in the hypothalamus posterior to the OVLT and dorsal to the ME may represent the paraventricular organ (PVO; Fig. 3h, j). The SCO, ME, OVLT, and PVO are CVOs of zebrafish—specialized areas of the blood-brain-CSF barriers involved in the communication between the brain, CSF, and blood (Tsuneki 1986; Joly et al. 2007; García-Lecea et al. 2017). All domains associated with the vIII are connected by expression in ependymal cells lining the ventricle (Fig. 3h, j). *camel* is still expressed in all these regions at 48 hpf.

**Fig. 4:**
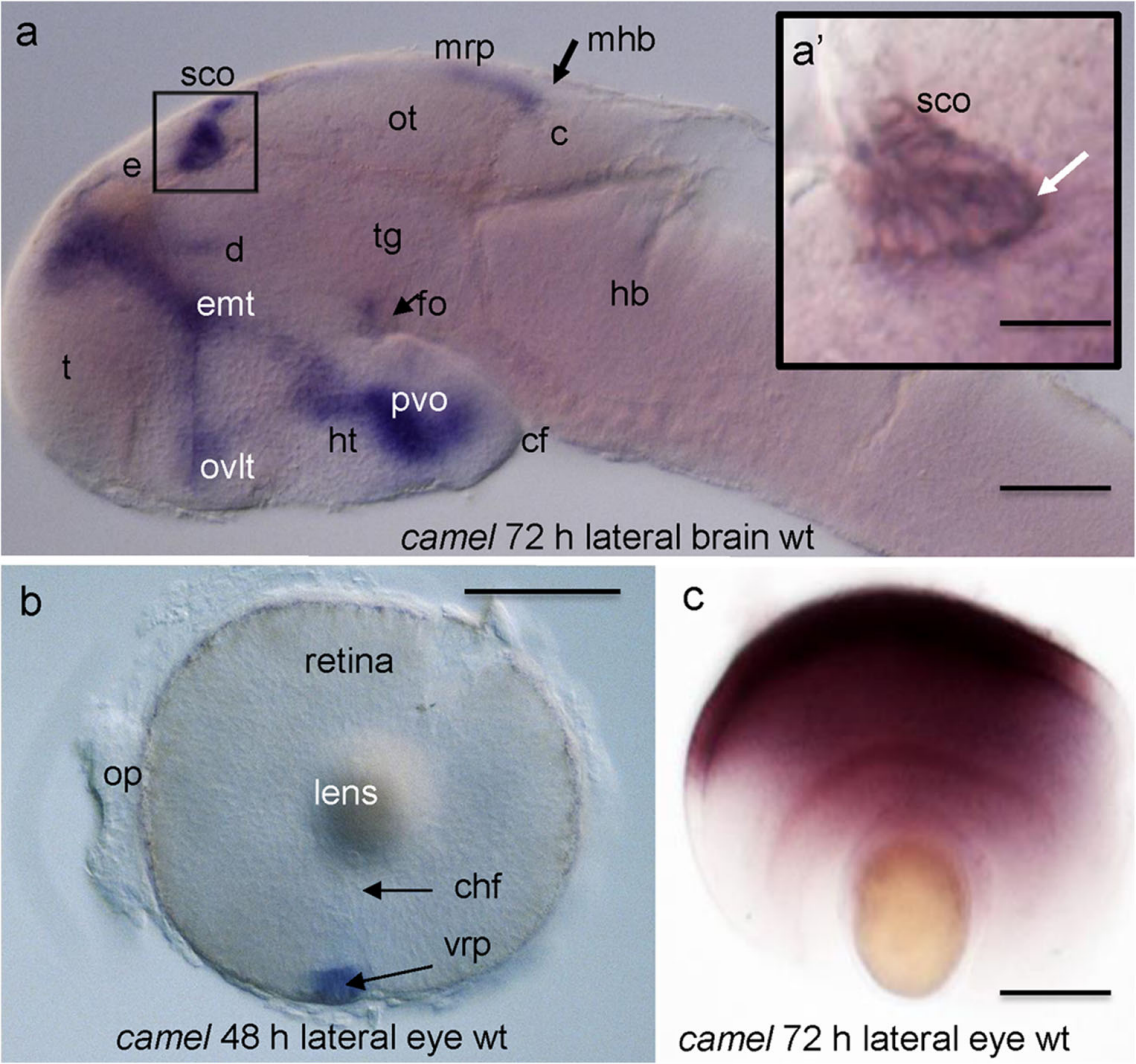
The expression pattern of *camel* during late development. *camel* expresses in the brain ventricular system, circumventricular organs, ventro-rostral patch (vrp), and the eye-differentiating retina. All images are in the lateral view. **a** 72 hpf, wild-type larvae. **b** 48 hpf, the left-hand side eye of the wild-type embryo. **c** 72 hpf, the left-hand side eye of the wild-type larvae. Abbreviations: c, cerebellum; chf, choroid fissure; cf, cephalic flexure; d, diencephalon; e, epiphysis; emt, eminentia thalami; fo, flexural organ; hb, hindbrain; ht, hypothalamus; mhb, midbrain-hindbrain boundary; mrp, midbrain roof plate; op, olfactory placode, ot, optic tectum; ovlt, organum vasculosum lamina terminalis; pvo, paraventricular organ; sco, subcommissural organ; t, telencephalon; tg, tegmentum. Scale bar = 100 μm except a’, where it is 25 μm

At this stage, *camel* expression is clearly defined in the FP and its most anterior region, the FO. The most posterior midbrain RP (mRP) bends to form the midbrain-hindbrain boundary (MHB) (Fig. 3j). These expression domains remain during the post-embryonic period at 72 hpf (Fig. 4a). By that stage, the identical cuboid cells of the anterior mRP, first seen at 24 hpf, become elongated (36–48 hpf) and form the triangular iron-shaped pocket of cells folded in the middle (Fig. 4a’, arrow). It looks like the mid-part of this cluster is being pulled towards posterior. Here, *camel* expression is more intense compared to the rest of the mRP. At 48 hpf, *camel* retinal expression is restricted to the ventro-rostral patch known as a site of initiation of retinal differentiation (Fig. 4b) (Larison and Bremiller 1990; Korzh et al. 1998; Neumann and Nusslein-Volhard, 2000). As differentiation of retinal cells progresses at 72 hpf, the *camel* expression domain covers most of dorsal retina (Fig. 4c). Other regions of expression at 48 hpf are represented by the heart (not shown). Hence, during neural development, the expression of *camel* takes place in several structures associated with the BVS.

### Camel isoforms differ at the level of the most C-terminal fibronectin type III domain

**Fig. 5:**
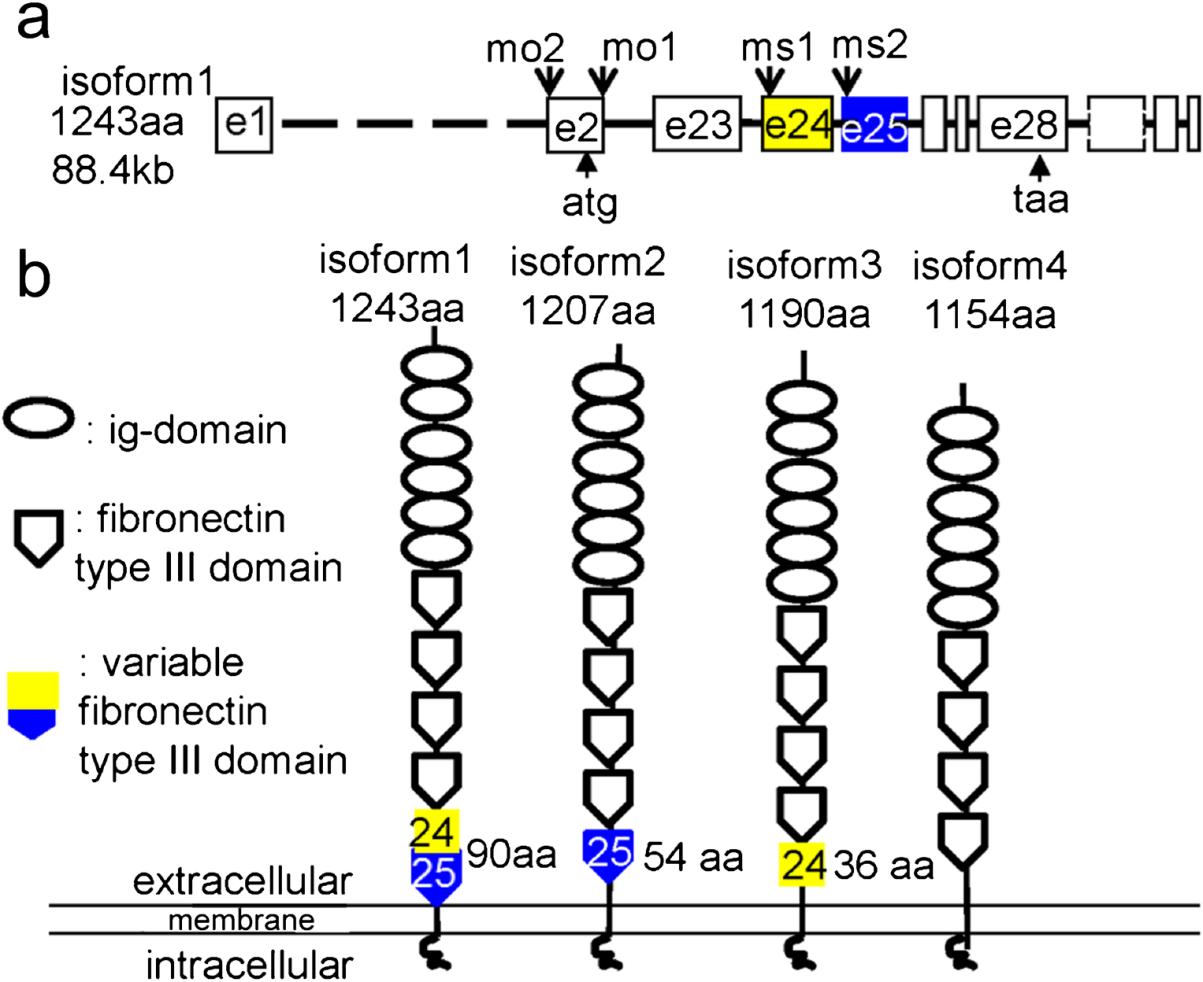
The schematics of organization of the *camel* genomic DNA and four differentially spliced isoforms. The putative proteins encoded by these mRNA isoforms vary at the level of the fourth fibronectin type III domain encoded by exons 24 and 25. **a** Organization of *camel* genomic DNA showing the target sites for morpholino and color-coded exons 24 (yellow) and 25 (blue). **b** Organization of the putative *camel* isoforms. The two halves of the sixth fibronectin type III domain are color-coded according to **a**. The Camel domain structure is generated using this software (http://expasy.org/prosite)

The variation in the cDNA sequence suggested that several isoforms of *camel* may exist due to differential splicing. We cloned them by PCR and found four *camel* transcripts that vary due to differential splicing of exons 24 and 25 (Fig. 5a, Suppl. Fig. 1) encoding the most C-terminal fibronectin (FN) type III domain (VIth). This may result in proteins with or without the whole domain or presence of one of its two halves (Fig. 5b).

### *camel* regulates cell adhesion in vitro

Camel belongs to a superfamily of L1-CAM-related molecules implicated in cell adhesion (Crossin and Krushel 2000; Maness and Schachner 2007). In human, L1-CAM LOF mutations cause X-linked hydrocephalus (OMIM #307000), and deficiency of this gene in *Xenopus* and mice also causes hydrocephalus (Rolf et al. 2001; Date et al. 2019). In human, CHL1 deficiency has been linked to cancer (Senchenko et al. 2011). Being a member of the class of neural CAMs, Camel may play a role in cell adhesion during development. To check this idea, the “hanging drop” assay (Steinberg and Takeichi 1994; Redies 2000; Fong et al. 2005) has been performed in combination with the MO-mediated knockdown of Camel. The pan-Camel MO (MO1–2) (Fig. 5a, Suppl. Fig. 2) and MO complementary to different regions of *camel* mRNA, including those blocking specific isoforms (MS1–2), have been used (Fig. 5a).

**Fig. 6:**
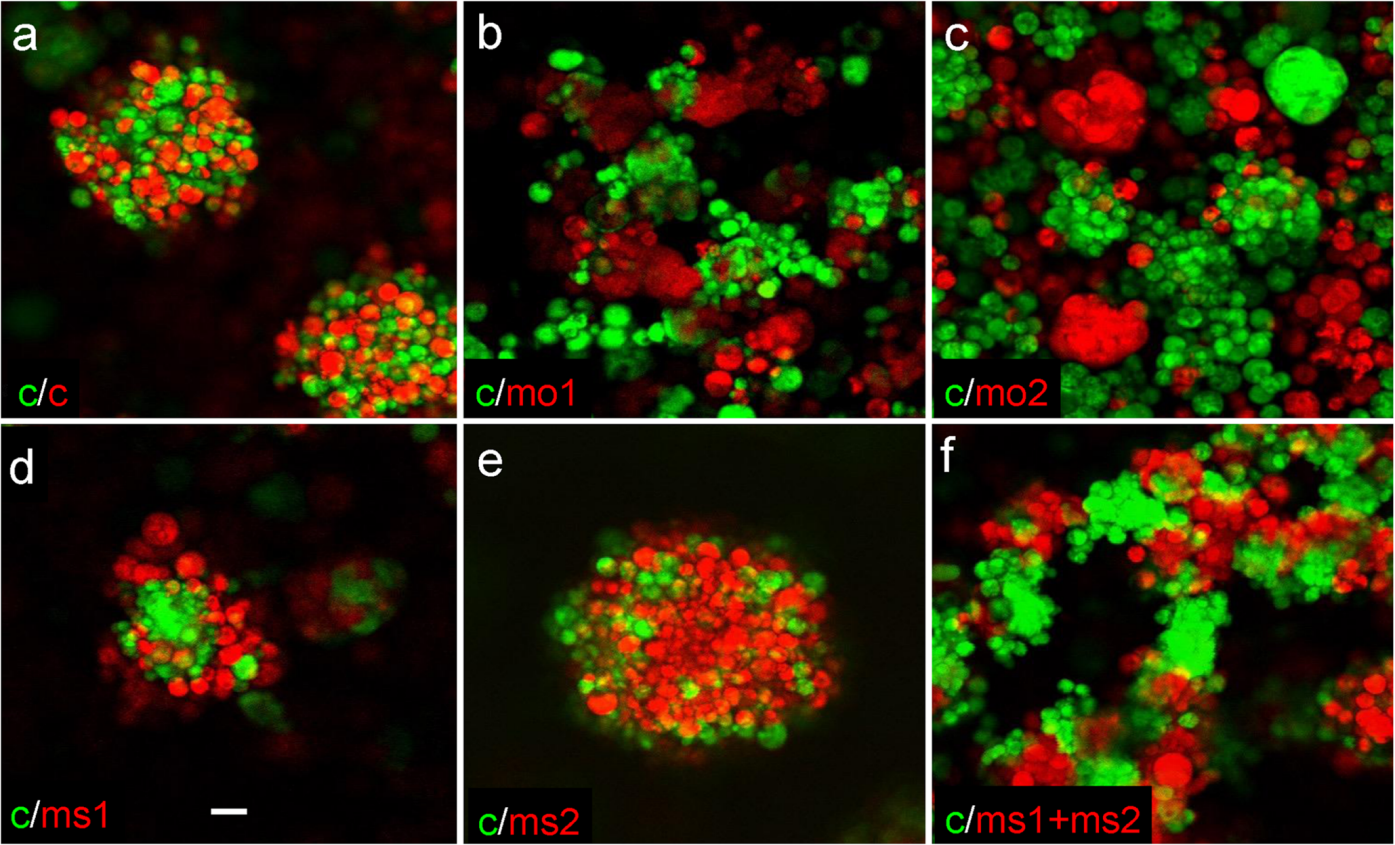
**a**–**f** Camel regulates cell adhesion in hanging drops. The control cells of different-color formed clusters mixed uniformly, unlike those in experimental conditions when Camel expression was affected in red cells by the anti-Camel morpholino. c (red, green), control; MO1 and MO2, pan-Camel morpholino; MS1, isoform 1,3-specific morpholino; MS2, isoform 1,2-specific morpholino. The combination of MS1 + MS2 blocks isoforms 1–3. Scale bar = 20 μm

The two different color dextrans were injected into embryos: one of the two groups of embryos used as a control and the second group co-injected with different anti-Camel MOs. The cells were isolated at 6-hpf stage, dissociated, mixed in equal proportion, and cultivated in hanging drops overnight. The control cells of two different colors formed uniformly mixed clusters (Fig. 6a). In contrast, when the pan-Camel MO (MO1 or MO2) was used and morphant cells (red) were mixed with control ones (green), the two types of cells formed distinct clusters of different colors (Fig. 6b, c, f) in the indication of different types of cell adhesion.

Distribution of cells caused by the isoform-specific MOs differs. MS1 blocks isoforms 1 and 3, which might leave isoforms 2 and 4 intact. MS2 blocks isoforms 1 and 2, leaving isoforms 3 and 4 intact. A combination of MS1 and MS2 targeted isoforms 1–3, leaving only isoform 4 intact (Fig. 5a, b). With single MO, the cells of one color formed the core and cells of another color attached at the periphery (Fig. 6d, e) in an indication of the different adhesion levels. This suggested a specific role for isoforms 2 and 3 encoding proteins containing different parts of the variable fibronectin type III domain (Fig. 5). The combination of MS1 and MS2 showed a phenotype similar to that of pan-Camel MO (Fig. 6f). Hence, in early development, Camel regulates cell adhesion. Its specificity may depend on the modification of the C-terminal fibronectin type III domain.

The FGF signaling plays a role in cell adhesion during development (Ben-Hur et al. 1998; Gallegos et al. 2019). Direct CAM-FGFR interaction has been suggested (Doherty and Walsh 1996) between the FN3 domains I–V of the similarly organized L1-CAM and FGFR1 (Kulahin et al. 2008). Possibly, a variation in the FN3 domain VI of Camel may affect this potential interaction.

### Camel loss-of-function in vivo

**Fig. 7:**
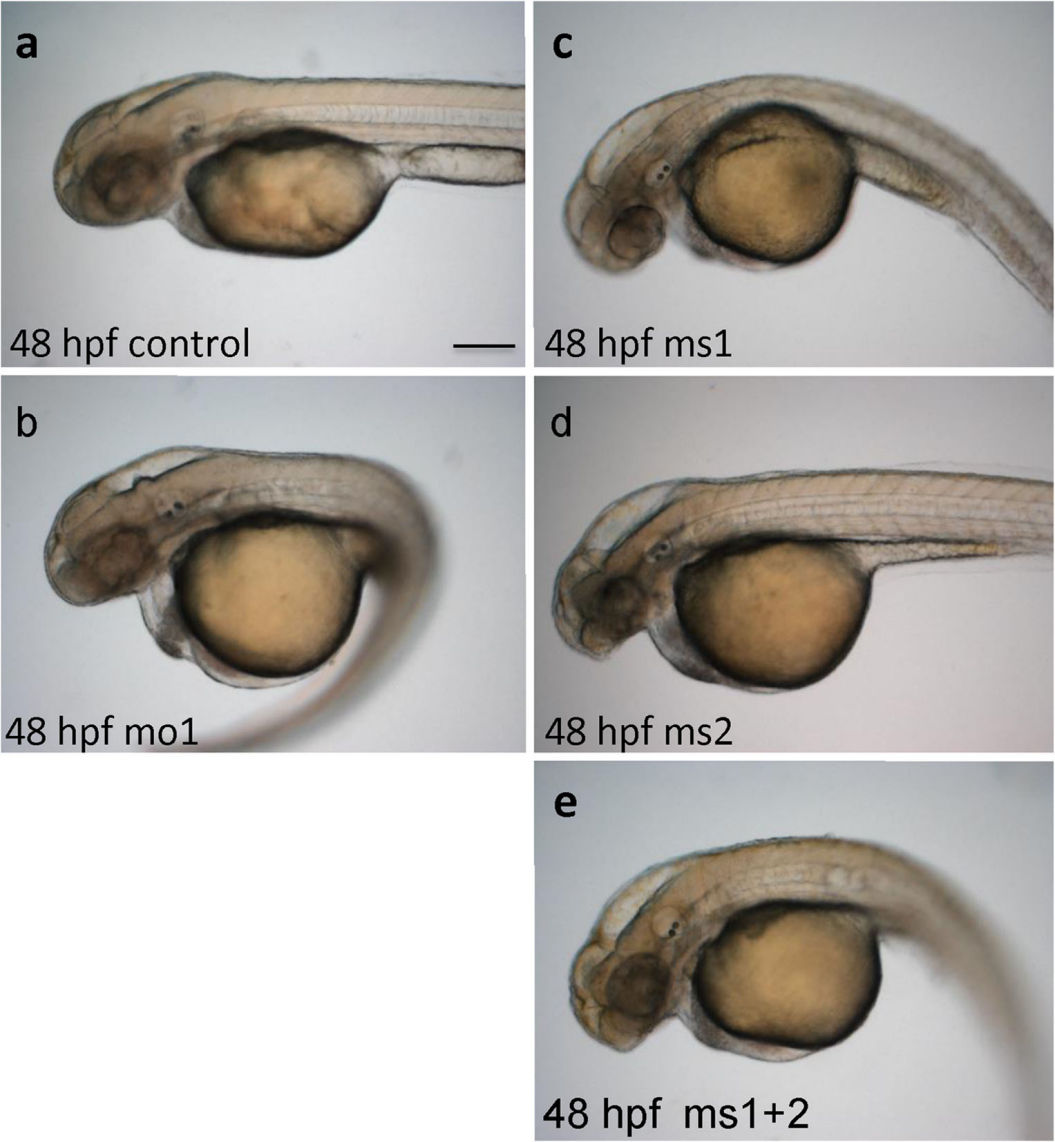
**a**–**e** The effect of different anti-*camel* MOs varies depending on the target. MO1 targets all isoforms causing the most severe effect, including the hydrocephalus, curled trunk, and edema. MS1 and MS2 target specific mRNA isoforms (1 + 3 and 1 + 2, correspondingly). MS1 causes the hydrocephalus and mildly curled trunk. MS2 causes hydrocephalus. The effect of MS1 and MS2 is additive in enhancing the trunk curvature. 50 embryos were observed in each experiment repeated three times. The phenotypes representing 95% of experimental animals were selected. Scale bar = 50 μm

To address a function of Camel during development, we microinjected the 1–2 cell-stage zebrafish embryos with the antisense oligomers (MO) against different regions of *camel* mRNA, including those blocking specific isoforms (see below), and raised them to different developmental stages. The pan-anti-Camel MO (MO1) caused a dose-dependent deficiency of the brain, including hydrocephalus, and curled trunk (Fig. 7b, Table 1). This phenotype has been defined previously as a hallmark of deficiency of CSF flow (Grimes et al. 2016; Boswell and Ciruna 2017; Sternberg et al. 2018). The isoform-specific MOs have a mild effect (Fig. 7c, d, Table 1), which increased when these MO were combined (Fig. 7e).

**Table 1:** Morphant phenotype

	MO1 (*n* = 48/50)	MS1 (*n* = 45/50)	MS2 (*n* = 42/50)
Brain	Reduced, hydrocephalus	Reduced, hydrocephalus	Reduced, hydrocephalus
Eye	Reduced	Reduced	Reduced
Trunk	Curly	Slightly curly	Straight

Early and new evidence strongly indicates that straightening of the body axis is RF-dependent (Kondrychyn et al. 2013; Cantaut-Belarif et al. 2018; Troutwine et al. 2020; Vesque et al. 2019, unpublished). Therefore, our findings that the deficiency of CHL1-related Camel causes the phenotype in the developing zebrafish usually associated with scoliosis support an idea of Sharma et al. (2011) that a deficiency of *CHL1* may be linked to adolescent idiopathic scoliosis.

### *camel* regulates formation of BVS in vivo

**Fig. 8:**
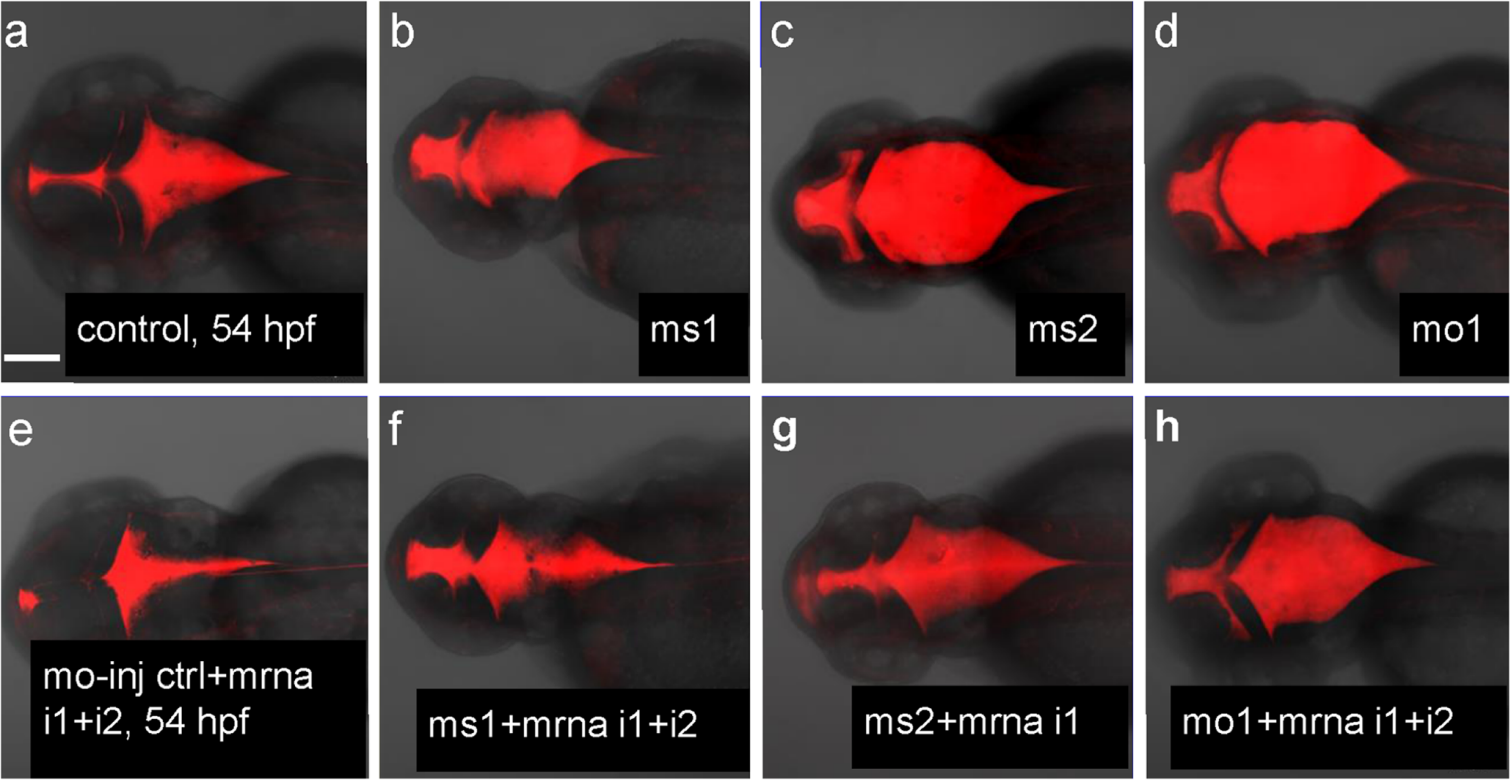
Rescue of hydrocephalus in Camel morphants by *camel* mRNA. **a** Control. **b**–**d** Morphants with brain ventricles filled-in by Dextran-Texas Red. **e** Control wild-type embryo injected by *camel* mRNA. Notice that the size of the ventricular system is reduced compared to the intact control. **f**–**h** Different morphants rescued by injection of different *camel* mRNA isoforms. All images are dorsal views of 54-hpf embryos with anterior to the left. Scale bar = 100 μm

To compensate for the inhibitory effect of MO, mRNA overexpression was used in the phenotype-rescue experiment. We focused on hydrocephalus, a phenotype that is easy to recognize and score (Shen et al. 2016). Different combinations of anti-pan-Camel and isoform-specific Camel MO and mRNA encoding different *camel* isoforms were injected (Fig. 5). As expected in all cases, the embryos injected with the anti-Camel MO (*camel* morphants) developed hydrocephalus (Fig. 8a–d), whereas mRNA compensated this effect to a different degree in an indication of specificity of MO effect (Fig. 8f–h).

Interestingly, Camel GOF in control embryos resulted in the reduced BVS (Fig. 8a, e). This added evidence to support the role of *camel* in maintaining tissue integrity during the formation of BVS by increasing cell adhesion. While this was not unexpected given the role of cell adhesion in the formation of the neural tube (Maness and Schachner 2007; Fame et al. 2016; Shen et al. 2016), it raised a question of how Camel regulates the BVS development.

### *camel* and the Reissner fiber

*camel* expresses in the SCO, FO, and FP, i.e., structures participating in the formation of the RF by continuously secreting into the CSF proteins that aggregate into microfilaments that become densely packed in a single fiber (Muñoz et al. 2019). The ever-growing RF extends from the SCO through the whole length of the spinal cord. A deficiency of RF has been linked to hydrocephalus (Meiniel et al. 2008; Jiménez et al. 2001; Wagner et al. 2003). Hence, we decided to explore a connection between *camel*, SCO, and RF. The AFRU antibody recognizes SCO-spondin (Rodríguez et al. 1984; Nualart et al. 1998), a product of SCO, FO, and FP. It specifically detects the RF, which polymerizes into several microfilaments in turn, assembled into a single thread-like structure of RF (Sterba 1969; Rodríguez et al. 1992).

**Fig. 9:**
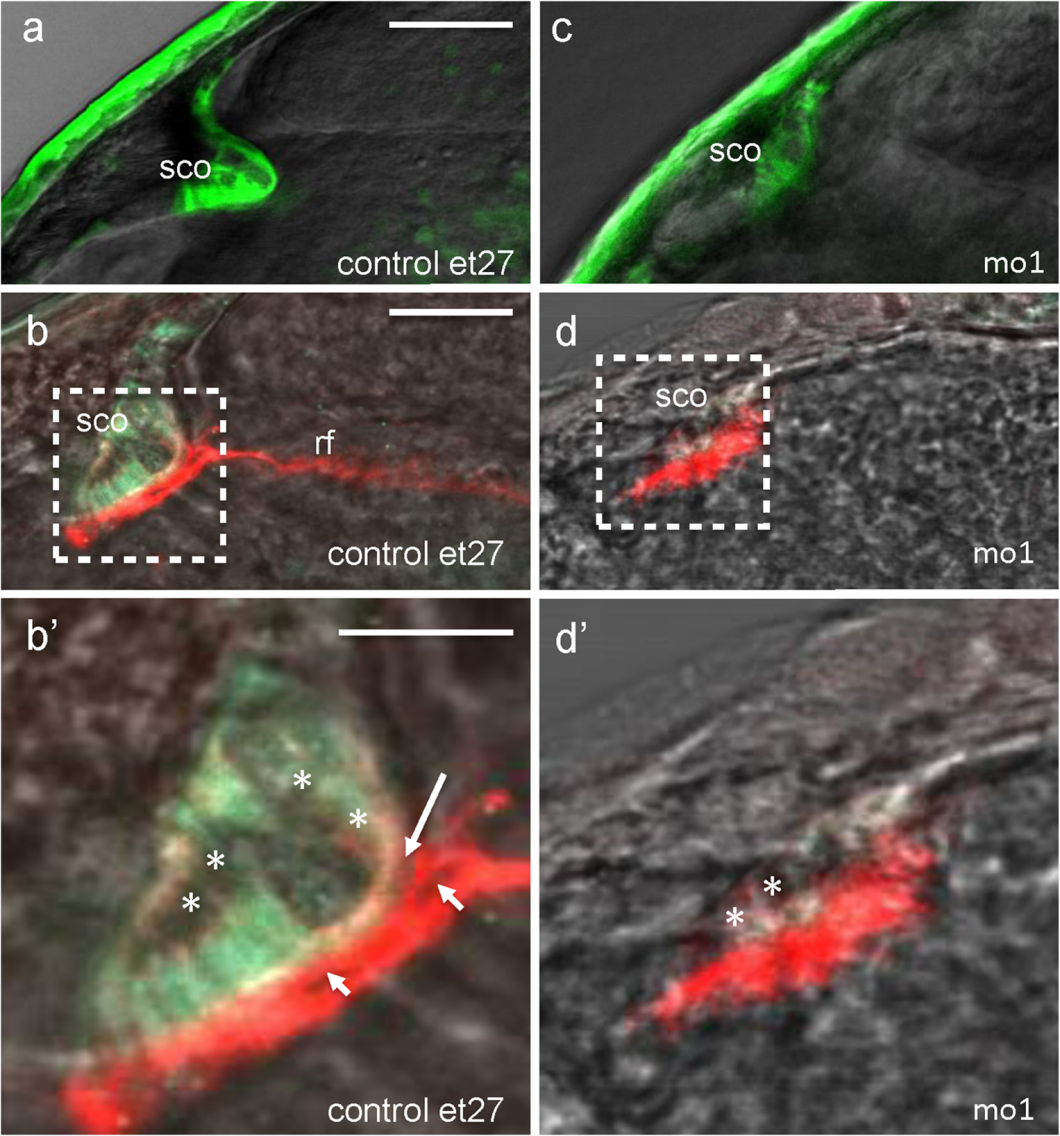
Camel required for the development of SCO and formation of Reissner fiber. **a**, **b** Control. **c**, **d** Pan-anti-Camel morphants (MO1). All images are of ET27 transgenics at 48 hpf. **a**, **c** In vivo images of GFP expression. **b**, **d** Double immunostaining for GFP and Reissner fiber. **b’**, **d’** Blowup of boxed areas in **b** and **d**, respectively. **b’** The AFRU staining in controls is mostly presented by the filamentous extracellular material with gaps between SCO and AFRU material (arrow) and in the AFRU material (arrowhead). Some faint AFRU staining was detected in the cytoplasm (asterisk). **d’** In morphants, the AFRU material is disorganized; it covers the SCO more with some residual signal in the cytoplasm (asterisk). Scale bar = 100 μm (**a**–**d**) and 50 μm (**b’**, **d’**)

The ET27 transgenics (Parinov et al. 2004; Kondrychyn et al. 2009; García-Lecea et al. 2017) express GFP in the SCO (Fig. 9a). When the control ET27 embryo is stained with AFRU antibodies, the signal can be detected at the SCO (Fig. 9b). This AFRU+ material is fibrous in controls (Fig. 9b’), consistent with prior observations in other species (Rodriguez et al. 1986, 1987b).

When injected with the pan-anti-Camel morpholino (MO1), the SCO of morphants fails to develop its characteristic iron-like shape (Fig. 9c). In contrast to controls, the RF in morphants fails to form, whereas the dotted and amorphous signal can be detected at the SCO (Fig. 9d, d’).

**Fig. 10:**
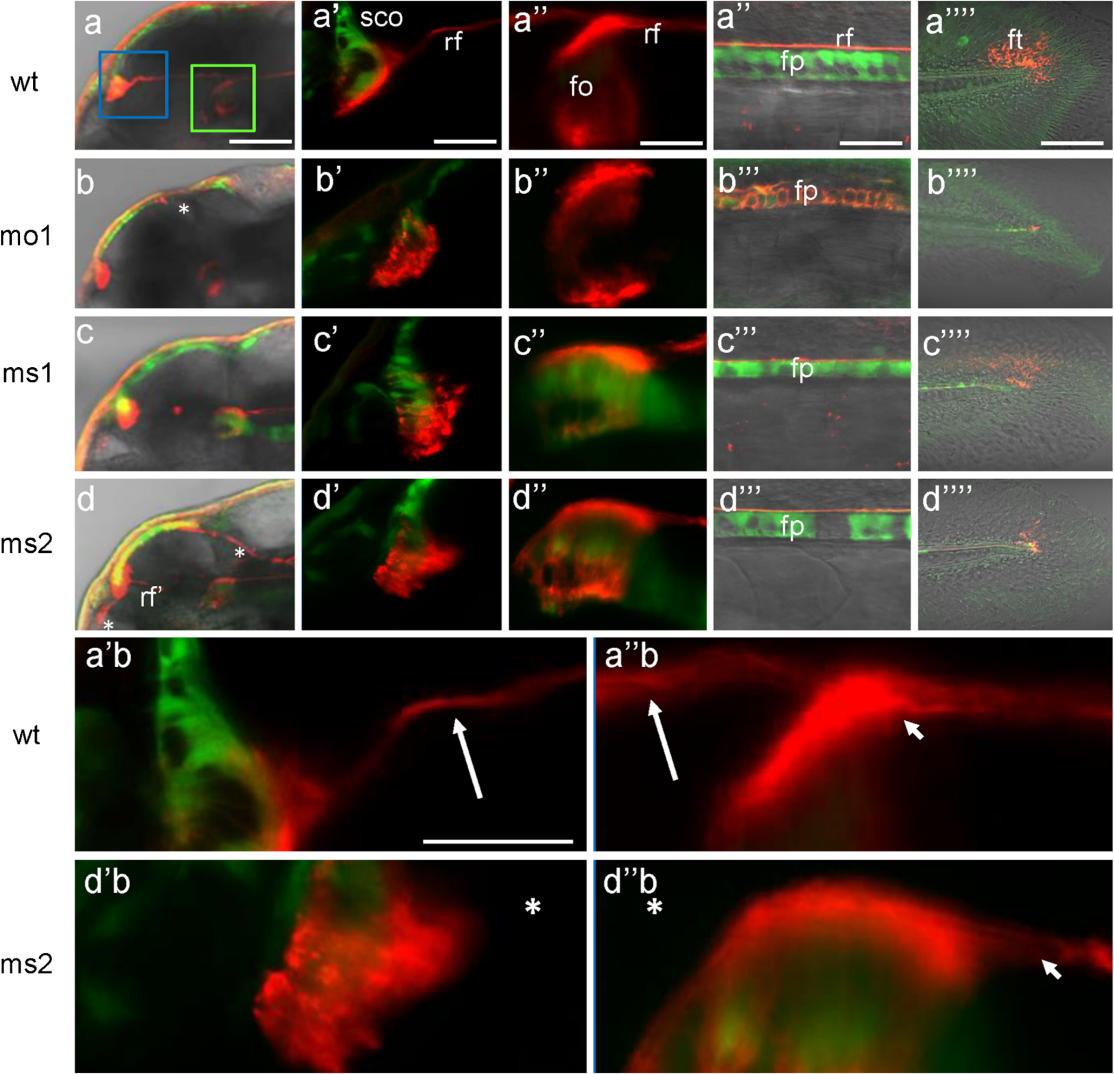
Pan-Camel and isoform-specific anti-Camel morpholino–mediated LOF differentially affects the formation of RF. **a** Being generated by the SCO (green, GFP, Tg(ET33-mi2a) and the RF (detected by anti-AFRU antibody, red) and spanning the BVS. It may obtain additional contributions from FO and FP. It extends through the central canal to the posterior end of the spinal cord to be disassembled at the filum terminale. **b** MO1 blocks the formation of RF (**b’**) and causes the distortion in the distribution of RF+ material in FO (**b”**) and FP (**b”’**). **c**, **d** In comparison, the effect of isoform-specific morpholinos MS1 and MS2 is less obvious. All confocal images of Tg(ET33-mi2a) transgenic 48-hpf embryos are shown as lateral views with anterior to the left. **a** Stack of sections. **a’** Selected sections to illustrate the primarily apical distribution of the RF material in the SCO. **a”’** and **a””** are the same as **c** and **d** shown for comparing the effect of MO. **a’b** Blowup of **a’**. **a”b** Blowup of **a”**. **d’b** Blowup of **d’**. **d”b** Blowup of **d”**. Asterisks indicate lack of RF, and arrows and arrowheads indicate RF. Abbreviations: fo, flexural organ; fp, floor plate; ft, filum terminale; rf, Reissner fiber; sco, subcommissural organ. Scale bar = 200 μm (upper left-hand side column, **a**–**d**), 100 μm (four other upper columns marked ’ to ””), and 50 μm (blowups)

The RF could be formed not only by secretion from SCO but also with an additional contribution of the FO and FP (Olsson 1956; Yulis et al. 1998). To be able to observe these structures, we repeated the anti-RF staining in the Tg(ET33-mi2a) transgenics with GFP expression in the SCO, FO, and FP (Kondrychyn et al. 2009, 2011). This staining demonstrated the presence of the RF+ material on the apical surface of the SCO (Fig. 10a, a’), FO (Fig. 10a, a”), FP (Fig. 10a”’), and filum terminale (Fig. 10a””). Following that, we repeated the MO-mediated Camel LOF in the Tg(ET33-mi2a) transgenics. An injection of the anti-pan-Camel MO (MO1) changed the shape of SCO and inhibited the formation of RF in the Tg(ET33-mi2a) transgenic morphants (Fig. 10b–b””). This change may happen because of the deficient secretion of SCO-spondin or absence of galectin-1 and clusterin that were suggested to regulate an assembly of the RF (Muñoz et al. 2019). In the SCO, FP, and FO of morphants, the amount and intracellular and extracellular distributions of RF+ material were abnormal (Fig. 10b’, b”, b”’). The AFRU+ material in the filum terminale was barely noticeable (Fig. 10b””).

The isoform-specific morpholinos MS1 and MS2 partially inhibited the formation of RF (Fig. 10c–c””, d–d””), indicating that secretion of the RF+ material depends on the combination of several *camel* isoforms. At the same time, the phenotypes caused by MS1 and MS2 differ. MS1 caused a general reduction of AFRU staining without affecting the distribution of RF+ material (Fig. 10c–c””). Whereas MS2 not only caused a general reduction of staining, RF material was found in the anterior vIII and associated with the mRP (Fig. 10d–d””); i.e., the RF+ material was significantly redistributed. These results demonstrated that Camel LOF affects the formation of RF.

### Overexpression of *camel* affects the distribution of RF+ material

**Fig. 11:**
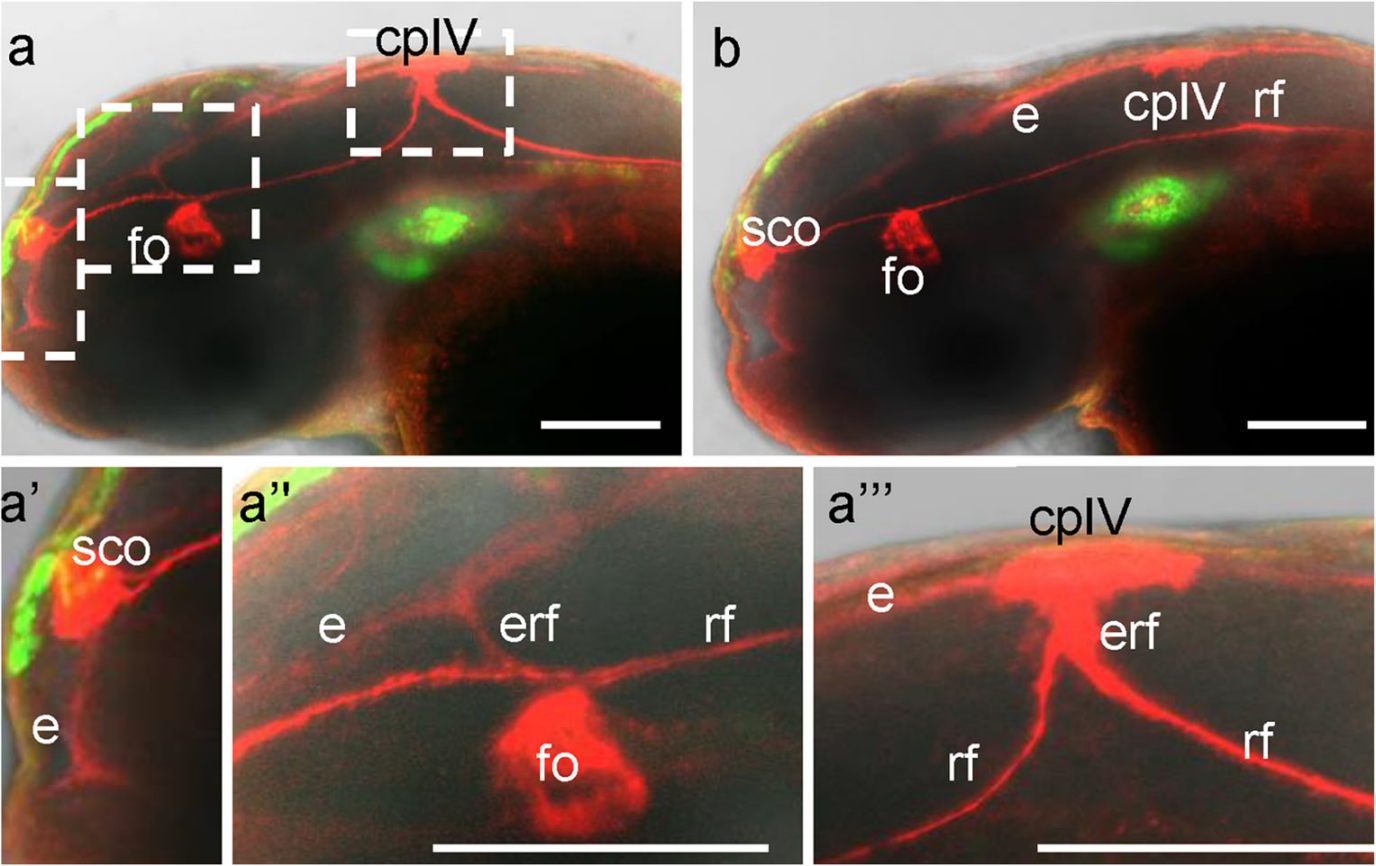
The overexpression of *camel* affects the development of the Reissner fiber. Upon *camel* overexpression, the RF+ material seems to increase in the BVS with its significant redistribution towards the choroid plexus of the fourth ventricle (CPIV) and ependyma (e, anterior and posterior of the SCO). The ectopic sprouts of RF (erf) in the Sylvius aqueduct (sa) formed. In the vIV, the RF changes the ventral trajectory (along FP) towards the CPIV in the dorsal position. In the posterior vIV, the trajectory of RF normalizes and leaves the vIV via the central canal. All images are lateral views of Tg(ET33-mi2a) transgenic 48-hpf embryos with anterior to the left. **a**, **b** Larvae after strong overexpression of Chl1a. Boxes in **a** represent SCO, FO, and CPIV and correspond to **a’**–**a”’**. Scale bar = 100 μm

**Fig. 12:**
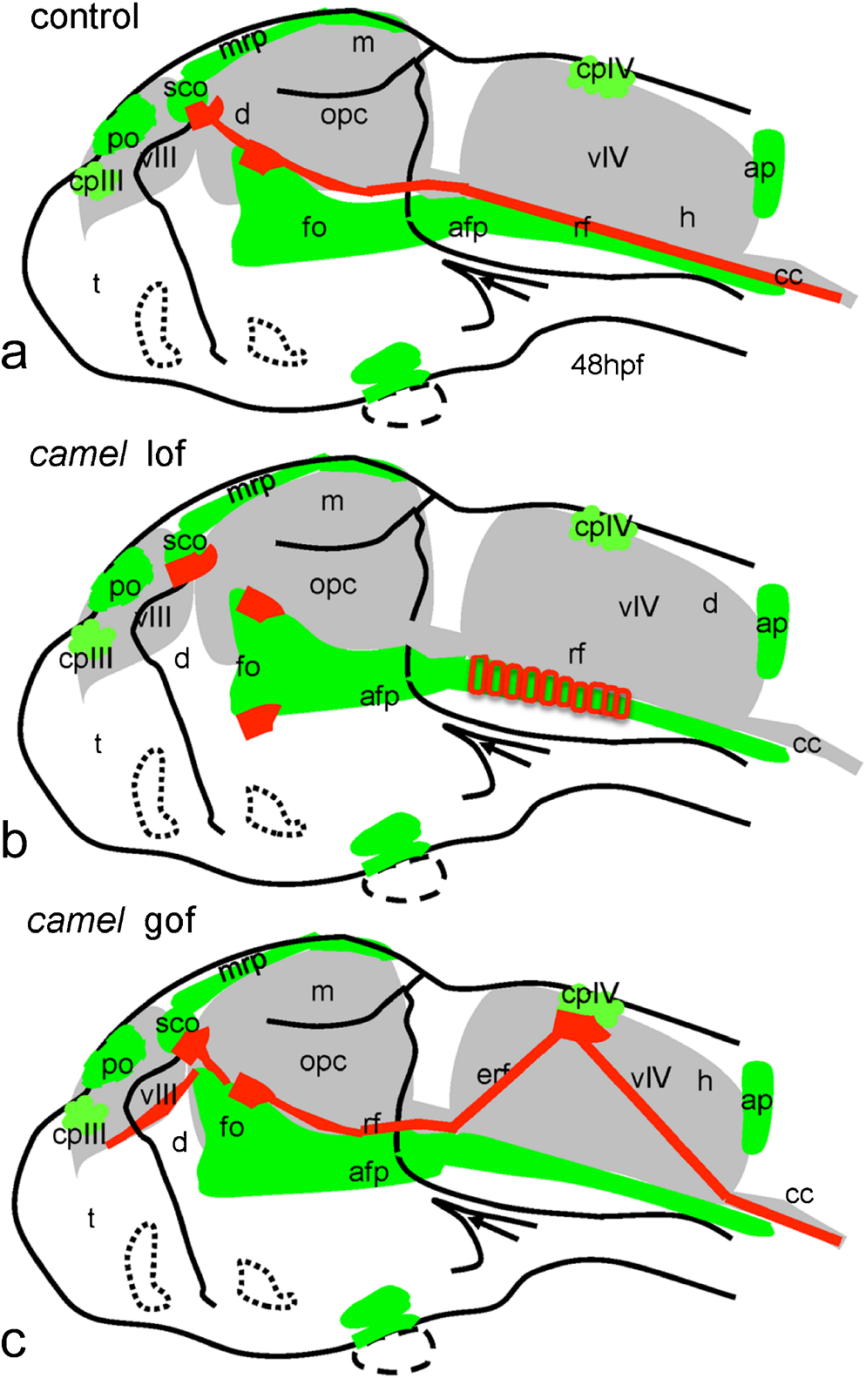
Schematics show organization of the Reissner fiber in respect of the ventricular system (based on Figs. 9, 10, and 11). **a**–**c** 48 hpf. **a** Controls. **b** Anti-Camel morpholino–mediated loss-of-function. **c** Camel mRNA–mediated gain-of-function. Green color, midline structures and some CVOs; red, RF and AFRU+ material. Abbreviations: afp, anterior floor plate; ap, area postrema; cc, central canal; cpIII, choroid plexus of the third ventricle; cpIV, choroid plexus of the fourth ventricle; d, diencephalon; e, epiphysis; erf, ectopic Reissner fiber; h, hindbrain; fo, flexural organ; fp, floor plate; m, midbrain; mrp, midbrain roof plate; opc, optocoele (Sylvius aqueduct); po, pineal organ; rf, Reissner fiber; sco, subcommissural organ; t, telencephalon; vIII, third ventricle; vIV, fourth ventricle

Thus, Camel LOF causes a defect of RF, which correlates with hydrocephalus. Camel gain-of-function (GOF) rescues hydrocephalus. Therefore, we checked the effect of *camel* mRNA microinjection, which causes the pan-embryonic Camel GOF, on the development of the BVS. An excess of RF material makes it challenging to ascertain the shape of SCO at 72 hpf (Fig. 11). The trajectory of anterior RF between SCO and FO is no longer straight. In the mildly affected larva, the RF+ material is in ectopic locations, the apical surface of the dorsal midline, and choroid plexus of the fourth ventricle (CPIV) (Fig. 11h). In the severely affected larvae, the additional sprouts of the RF are dorsad of FO. The RF connects to the CPIV. Posteriorly, the RF retakes its route alongside the FP and enters the central canal of the spinal cord (Figs. 11g and 12b). An ectopic RF material covers the surface of ependyma in vIII and vIV. The Camel LOF and GOF experiments demonstrated that the formation of RF depends on Chl1-related Camel.

## Discussion

The deficiency of the CSF flow has been linked to the RF and motile cilia abnormalities resulting in hydrocephalus and scoliosis (Cifuentes et al. 1994; Perez-Figares et al. 2001; Lang et al. 2006; Grimes et al. 2016; Boswell and Ciruna 2017; Cantaut-Belarif et al. 2018). Juvenile scoliosis develops in correlation with destabilization of the RF (Troutwine et al. 2020; Vesque et al. 2019, unpublished). *Chl1* has been suggested as a susceptibility gene of adolescent idiopathic scoliosis (Sharma et al. 2011), although this idea remains short on supporting evidence (Qiu et al. 2014). Our study demonstrates the role of *camel* in the development of the BVS, formation of the RF, and straightening of the body axis, which has been linked with the proper development of axial structures and, in particular, the RP (Kondrychyn et al. 2013; Korzh 2018). And, it provides experimental evidence to support an idea that Chl1 could be a candidate gene for idiopathic scoliosis.

In the zebrafish model of scoliosis, there is a failure to straighten the body axis during development (Grimes et al. 2016; Cantaut-Belarif et al. 2018; Troutwine et al. 2020; Vesque et al. 2019, unpublished). During development, the curly tail of zebrafish embryo straightens up at 16–30 hpf and the axial structures (FP, RP, and HC) experience the elastic deformation (Lowery and Sive 2009; Kondrychyn et al. 2013; Shen et al. 2016; Korzh 2018). Upon Camel LOF, embryos fail to straighten and develop extended BVS similar to that in the scoliosis model. This phenotype suggests a role for Camel in the development of the BVS and supports the idea that CHL1 is one of the candidate genes for scoliosis.

*camel* is not expressed in all ependymal cells lining the cavity of the BVS and central canal, but in the CVOs, where ependyma lacks cilia (Krisch et al. 1978; Gross and Weindl 1987). Hence, it is unlikely that motile cilia will be directly affected by Camel LOF. The hydrocephalus and scoliosis in *camel* morphants could be due to the abnormal development of CVOs and axial structures, where this gene expresses (Figs. 3 and 4; other data not shown). In particular, SCO, FO, and FP, involved in the formation of RF, seem to be affected in Camel LOF (Fig. 10). The defect of SCO causes hydrocephalus (Galarza 2002; Pérez-Fígares et al. 2001; Vio et al. 2000; Louvi and Wassef 2000). Still, a defect of signaling structures such as FP may affect the differentiation of target cells. An excessive RF material could stick either to the signaling or motile cilia, which incapacitates these structures indirectly and influences CSF flow (Fig. 10b). Hence, while cells with motile cilia seem do not express Camel, the indirect effect of Camel on their differentiation and/or function could not be ruled out.

The defects of RF cause hydrocephalus (Cifuentes et al. 1994; Lang et al. 2006; Vio et al. 2008). For example, in the *hyh* mice deficient in α-Snap, the RF is severely affected (Pérez-Fígares et al. 1998; Perez-Figares et al. 2001). This defect results in an ectopic accumulation of the RF material in BVS, which is reminiscent of deficient secretion and distribution of the RF material in *camel* morphants (Fig. 10). When RF is absent, the embryos fail to straighten their body (Fernández-Llebrez et al. 2001; Cantaut-Belarif et al. 2018). Similar to that, the Camel LOF results in the absence of the RF, hydrocephalus, and curly body (Figs. 7b, 8d, and 10b). These defects suggest that Camel acts in the formation of the RF.

Upon Camel GOF, the CPIV takes the role of an ectopic organizing center of RF that changes the RF trajectory (Fig. 11). Here, two possible scenarios could be at play: (i) an ectopic RF material derives from its natural sources (SCO, FO, FP) and, when in excess, sticks to CP, or (ii) it is produced by CP and ependymal cells. The two CVOs in question—the CP and SCO—may derive from the RP (Kiecker 2018; Korzh and Kondrychyn 2020). These CVOs are similarly affected by ectopic expression of En1 (Louvi and Wassef 2000). It seems the CP and SCO share enough common molecular mechanisms to trigger the ectopic production of RF material in CP. SCO-spondin is not expressed in the CP, unlike that in the SCO, although CP and SCO do share secretion of TTR (Montecinos et al. 2005), and GFP in the enhancer-trap line ET33-10 (Garcia-Lecea et al. 2017). The CP and SCO are similarly affected by ectopic expression of En1 in the RP that causes early cell death in this structure (Louvi and Wassef 2000).

On the other hand, it has been shown in mammals and birds that during normal development, first, disorganized RF material appears, and second, the RF forms due to the contribution of polymerizing factors (Hoyo-Becerra et al. 2005) with Clu and Lgals1 being the prime candidates for this role (Muñoz et al. 2019). We showed previously that in the developing zebrafish, Clu is expressed in the RP (Jiao et al. 2011; Jeong et al. 2014). The CP and SCO of zebrafish express *lgals2a* and *lgals2b* genes (Thijssen et al. 2006). Since Camel LOF inhibits the secretion of RF material in SCO and FP (Fig. 10a, b), an ectopic RF material likely derives from its natural sources (SCO, FO, FP) and, when in excess, sticks to the CPIV, which is one of the sources of factors involved in RF polymerization.

Camel as a member of the neural CAM family could be critical for adhesion of cells in the SCO, FO, and FP. The results of experiments in “hanging drops” taken along with observation of changes of the shape of SCO and FP in Camel LOF embryos confirm this notion (Figs. 6 and 10b”). The differential activity of its isoforms regulated by differential splicing at the level of one of the fibronectin domains seems to add another level of complexity in the regulation of cell adhesion in the BVS.

It seems that the CVOs are subjects of elastic deformation similar to the RP and FP (Kondrychyn et al. 2013). During development, the SCO transforms from a line of relatively short columnar cells into the iron-shaped pocket of elongated cells. Given that the SCO generates the RF and remains connected to it, perhaps, the CSF flow pulls the RF and attached SCO cells towards posterior, resulting in the morphogenetic change of the SCO. The results of the Camel LOF agree with this suggestion. In the absence of the RF, the morphant SCO remains rather flat (Figs. 9 and 10).

Here, we demonstrated that the Chl1-related CAM Camel plays a role during neural development by regulating cell adhesion in the axial structures of developing zebrafish, RP, FP, and the RP-derived SCO. Camel deficiency results in the failure of the development of RF, hydrocephalus, and scoliosis.

## Electronic supplementary material

ESM 1 (PDF 212 kb)

ESM 2 (PDF 29 kb)

ESM 3 (PDF 347 kb)
