# Supplementary figures and images for: Camel regulates development of the brain ventricular system
Source: Cell Tissue Res. 2020 Sep 9;383(2):835–52. doi: 10.1007/s00441-020-03270-1 (PMC7904751; doi:10.1007/s00441-020-03270-1)

**A**

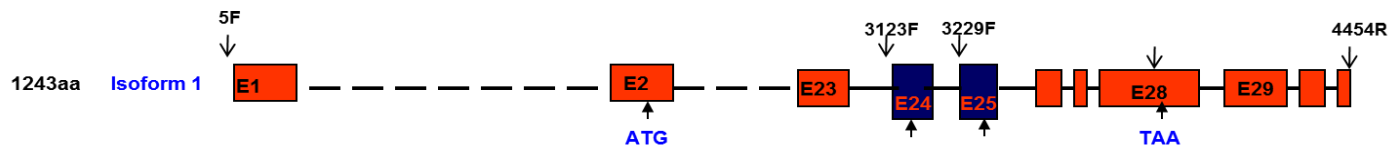

primers

1-cell

6h

12h

24h

48h

72h

144h

**B**

5F-2R

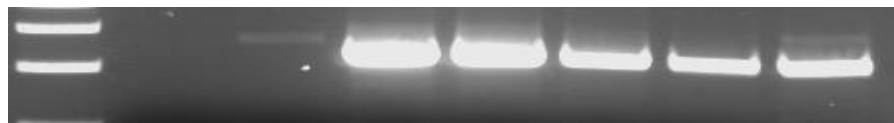

**C**

159F-2R

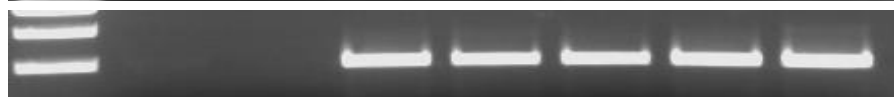

**D**

3438F-1R

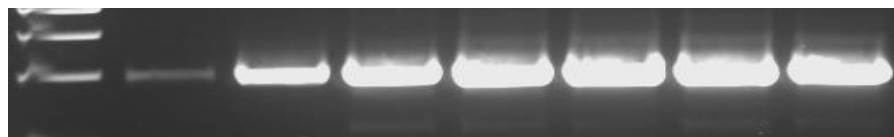

**E**

3035F-1R

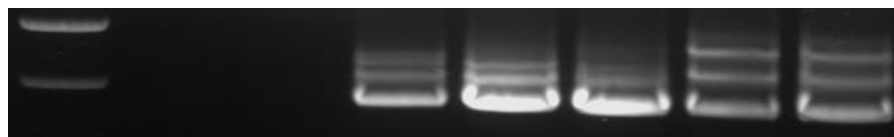

**F**

isoF-isoR

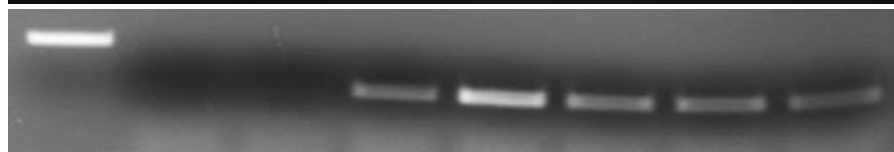

**G**

elf-1α

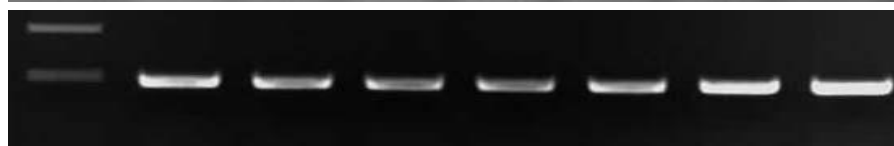

Suppl. Fig.1.

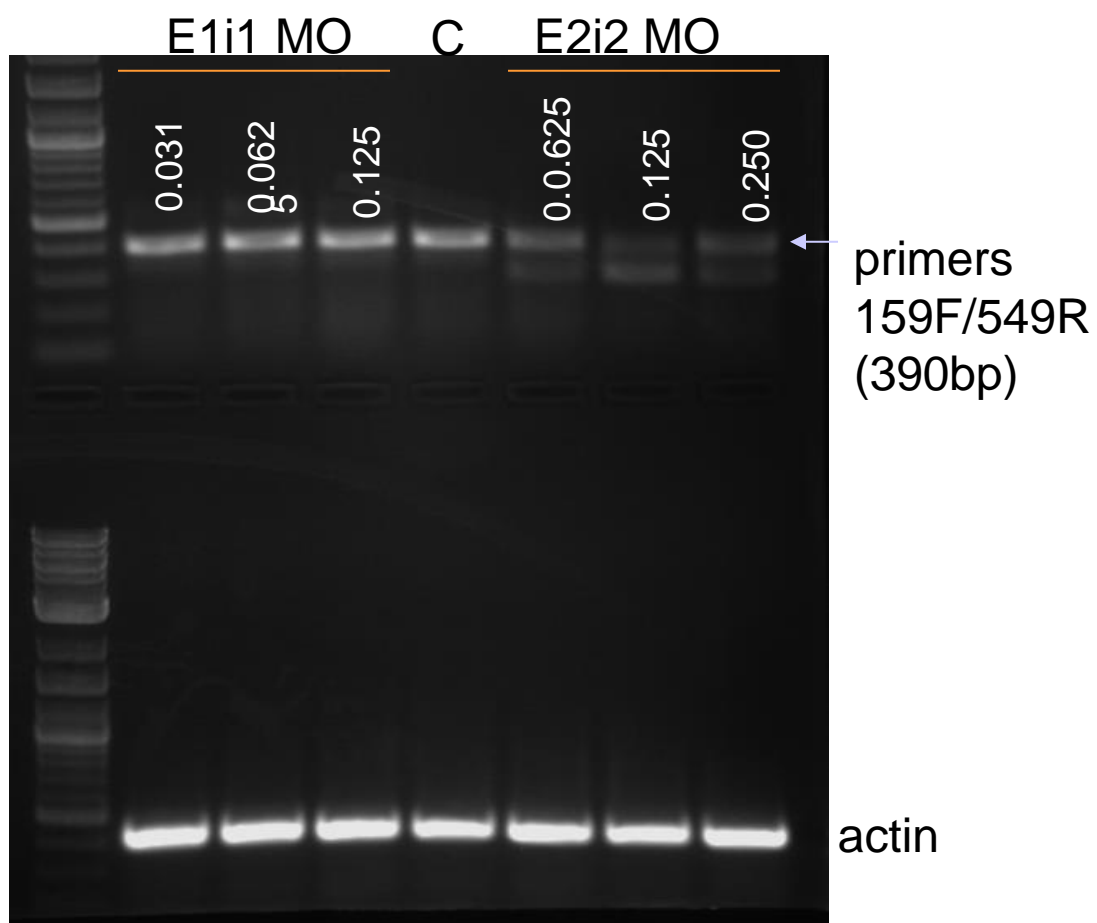

Suppl. Fig. 2.

Supplement: Supplementary file 1 — (PDF 212 kb) [file 441_2020_3270_MOESM1_ESM.pdf]
