# Supplementary material for: Camel regulates development of the brain ventricular system
Source: Cell Tissue Res. 2020 Sep 9;383(2):835–52. doi: 10.1007/s00441-020-03270-1 (PMC7904751; doi:10.1007/s00441-020-03270-1)
Supplement: Supplementary file 2 — (PDF 29 kb) [file 441_2020_3270_MOESM2_ESM.pdf]

Legends for Supplementary Figures

<u>Supplementary Figure 1.</u>
Panels A, B show the organisation of the isoforms 2, 3 and location of morpholino used for isoform-specific knockdown.
Panels C and D shows results of RT-PCR used to identify the mis-splicing caused by injection of Camel-isoform-specific MO (MS1 and MS2, respectively) or the standard morpholino (CK).
Each sample was from a pool of more than 50 embryos at 24 hpf.
The band 1 and band 2 in figure C were amplified by the primer pair P1/P2 [P1=2585F (5' AGGTGTTGAACTCTACACTGC 3'); P2=iso10R (5' TGCTAGAAGCTTTCCTGGTCT 3'). The band 1 and band 2 were cloned into T-easy Vector and sequenced. The sequencing results showed that after injection of MS1 the exon E24 (108 bp) was spliced out precisely.
The band 3 and band 4 in figure D were amplified by primer pair P3/P4 [P3=iso10F (5' TGCAGCATTCGTGCTCAACGT 3'); P4=3271R (5' AACAGCCGATGAGGACAAGCA 3'). The band 3 and band 4 were cloned into T-easy Vector and sequenced. The sequencing results showed that after injection of MS2 the exon E25 (162 bp) was spliced out precisely.

<u>Supplementary Figure 2.</u>
This is the isoform 1 cDNA sequence. The sequence in brown color is that of exon 24(E24), which is 108 bp in length. The sequence in green color is exon 25 (E25), which is 162bp. The underlined sequences correspond to the primers used to amplify the transcription products of morphants(CK, MS1, MS2) by RT-PCR. Between the P1 and P2 there are 868 bp.
Between the P1 and P2 there are 870 bp. Between the P3 and P4 there are 368bp.
