# Supplementary material for: Camel regulates development of the brain ventricular system
Source: Cell Tissue Res. 2020 Sep 9;383(2):835–52. doi: 10.1007/s00441-020-03270-1 (PMC7904751; doi:10.1007/s00441-020-03270-1)
Supplement: Supplementary file 3 — (PDF 347 kb) [file 441_2020_3270_MOESM3_ESM.pdf]

primers:

1F: 5’-TCTCTTGAAGGAGTCGGTAAG -3’

1R: 5’-TAGTCCTGCTGATCCTGTCAA -3’

2R: 5’-TGCACATCTTCACCATGCTGA -3’

5F: 5’-TGCTCATCTCTTCTGACAGGA -3’

3599R: 5’-TTGGGCCACTGTTAAGAAGTTC- 3’

3599R-XbaI: 5’ TGCTCTAGATTGGGCCACTGTTAAGAAGTTC -3’

3987R: 5’-AAGAACGGATTCCTGGAGTCA -3’

3564F: 5’-TGGACATACCCCAGGAACTTC- 3’

3438F: 5’- TGATCGCAGTGAGGATAGCTC -3’

518R: 5’-AACTTGGGTACCATAGGCACA -3’

159F: 5' -GAGCATATCCTTCATCTTTCTC- 3'

106F: 5’- TCATCTTTCTCTGGCTGGGAT- 3’

106F-ClaI: 5’-CCATCGATTCATCTTTCTCTGGCTGGGAT- 3’

78F: 5’ -TCCTGACGAGTGTTTTGAGCA- 3’

656R: 5’- CTCTCATTCTGCTCAATGTGC -3’

911F: 5’-AGCTGGAGTGTATTGCAGAAG -3’

1661R: 5’-AGTTCAGTGGTGATGCTTGCA -3’

1425F: 5’-TCTGGAGTATGCAACCATGTTG -3’

2028F: 5’-GAACACCAGTGATCACAACAG -3’

2585F: 5’-AGGTGTTGAACTCTACACTGC -3’

2816F: 5’-ACCGGAGAGGGAATGGGC -3’

2990F: 5’-ACCAAACAGTTGTAGATAATGAG-3’

3009F: 5’-TGAGACTCTGGGTGAGCTGAA-3’

3035F: 5’-TGAACATCTCTGGAGCAGACA- 3’

ISO10-F: 5’-TGCAGCATTCGTGCTCAACGT- 3’

ISO10-R: 5’-TGCTAGAAGCTTTCCTGGTCT -3’

ISOuni10-R: 5’-CAGATACCATCACGGTGGTTC- 3’

ISO8-F: 5’-ATGAACCACCGTGATGGTATC -3’

4394R: 5’-GAAGAACGGATTCCTGGACTC-3’

4369R: 5’-GATGTGCTAAAGGCTGCATTC-3’

4333R: 5’-TGGACCTGTCATGTCAGAAGC-3’

4298R: 5’-ACTCATGGCTGAGCAGAAGCA-3’

4263R: 5’-TGTACCTGGCATTTAGTGACC-3’

pE23E25-F: 5’-CTGCAGCATGTGATGGTATCT-3’

pE26E24-R: 5’-GTCTGCTAGAAGGGTGGTTCA-3’

3009F: 5’-TGAGACTCTGGGTGAGCTGAA-3’

2953F: 5’-GAAACCAACGGCCATCTCACAG-3’

3025F: 5’-CTGAACTCTGTGAACATCTCTGGA-3’

3426R: 5’-CTCTTCAAAAATGCTAGGGACACC-3’

3271R: 5’-AACAGCCGATGAGGACAAGCA-3’

The DNA sequence of camel isoforms

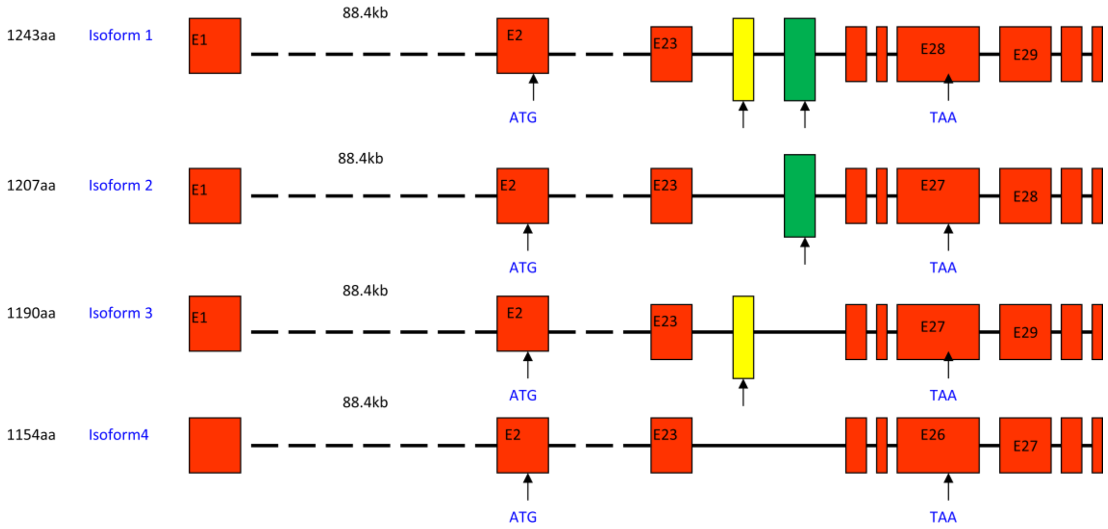

Isoform 1 cDNA sequence

ATTCTGCTCATCTCTTCTGACAGGAGCTTAACGCCGACTTAAATCAGATCTTTCTCTTGAAGGAGTCGG
TAAGAAGCTCCTGACGAGTGTTTTGAGCATATCCTTCATCTTTCTCTGGCTGGG**ATGAGGGGGCTCAG**
GAGTCTCAGTGTTGGGCTCCTGTCGGCCTTCTTCTCCACCACCTTTGGTCTTTATTTCCCTCCTCACGT
GGAGCAGCTGCCCACCATCACCGCTCAGACCTCAGGCACTATCATCGCCCTACCATATGATGAGCGAT
ACATATTCAAGTGTGAAGCCAAAGGAAACCCAAGACCTACGTTCCGCTGGACAAAAGATGGGATG
GATTTTGACCCACATAAAGACCCTCAGGTCACTACAGAAGAAAATACTGGAACCTTTGAGATACCA
AATAAAAAAGATAAAGTGAAATACCAGGGAAAGTACAGATGTTACGCCTCCAACAAACTCGGAAC
TGCAACTTCAGAAGAAGCCGAATTTATTGTGCCTATGGTACCCAAGTTTCCCAAGGAGAAGATAGA
GCCAATTGTGGTCACAGAGGGTGATTCGGTGGTTTTGGAGTGTAACCCGCCACAAGGGATTCCACC
CTGGCAGCTGTACTGGATGACCAATGATCTCCGGCACATTGAGCAGAATGAGAGGGTGTCCATGGG
CCTCAGTGGGAATCTGTACTTCTCTAATACGCTGGTGTCAGACAGTCATGATGACTACTGCTGCTTCG
CCTCCTTCTTCAAAATACGCACAATCGTCCAGAAACCCCGCATGGTCCTCAAGGTCAAGCCAGTTGA
TGCAAAGCAAGAGGATGGTGATGCCAAGATTGTTCAGGAGAGGAAGCCAAGCATCTTGGTTCCCT
CCAGTCATTCAACGACTTTCCTGAAAAAAGGAGAAGAGCTGGAGCTGGAGTGTATTGCAGAAGGA
CTGCCTACACCGGAGGTAGAATGGATCAAGATATGGGGTGACTTACCAAAGCGCACGCAGATAAAG
AACTTCGGAAAGCTTCTGACAATACCAAACATCAGAGAAGAGGACGAAGGAAAATACATGTGTAA
AGCCAAGAATCAGCATGGTGAAGATGTGCATCATTTCCAGGTCGAAGTAGAGGAGCCACCGAGCT
GGCAGAAGGAGCCGCTGAAGAGTCAGTTAGCATGGATTGGATCTGACATCCACATCAACTGTTCTG
CTACCGGAAAACCGCAACCCACAATCACCTGGAAAAGAAACGGCAAGCCCCTCGATGATTTCCCTT
CCACCCATCACAAAATGCTTGACGACAGAATAATCATACTCAAAGCGGAGCAGAAAGATACCGCTG
TGTACCAGTGTGAGGCCTCCAACAAACACGGCAGCCTACTGGCCAATGCTAATGTGCTAGTCATGAA
TCACCCTCCTTTGATTCTGACGAGGAATTATCTGGAGTATGCAACCATGTTGGGCAAGAGTGTGATT
ATGGACTGCAATGTCTTTAGTTCACCACCTGCCACCCTTAACTGGAGAAGAGAAGACCCAGAGGGC
TCTGTAGATGGAGAGAGATATTCTCTGCTGAAGAATGGCTCCCTGCAGATTCACAAAGTCGAGATG
GAAGATATGGGCCAGTACAAATGCTTGGCAAACAACTCCGAGGGCTCTGCAAGCATCACCACTGAA

CTTTTCATTAAAGATTCCACTAGGATCGTGGAGGCTCCTCAAGACATGAAGGTCATCCGGGGATCTG
TGGCCGAGCTGGAGTGCCAGGTGGAGTGCGACCCGTCTCTGAGAAGAGAGCTGGAGTTCGTTTG
GCTCAAAGATGGGATAAATATCCTCTCGAACTCCTCAGAAAGGTATTTTACAGATGAAGGAATTCTT
CAGATATTCAACGTGAGTCACAGTGATCAAGGGAATTACACCTGTATTGTTCGAACATCCCTGGACC
AAGATACAGCTTCTGCATACATAACAGTCTTAGATGTCCCCGATCCTCCGGTTGAATTATTGCTCAGT
GATCTGAAAGACCGCAGTGTGAAGCTCCACTGGGTGAACACCAGTGATCACAACAGTCCCATAACA
GAGTTCATCATCCAGTACGAAGAGAGCCACTGGGAGCCAGAGAAGTGGAAAGATCTTCTTCGAGT
GTCTAGCAGGAAGTTCTCAGCCCCCTTGGCTCTGTACTGCCACATTAACTACCAGTTCAGAGTCATCG
CTGTCAATGAAATAGGGAGAAGTCATCCCAGTATGCCCTCAGGAAGATATAAAACACCTCCATGTGC
TCCTGACAGGAACCCAGAAAACATTAAGATTGAGGGTCACCTGCCACATCAGATGGACATCAGCTG
GGAGCCGCTCTTGCCGGTGGAGCACAACGGTCCCGGTCTGGAATACAAACTGAGCTACAGGCTCCT
GGGTGTCGAGGACAGTTGGAAAGAGCAGATGGTAAAGAGACACTCTTTTGTGGTGCGAGACACC
CCGACATTCGTCCCCTATGAAGTCAAAATCCAGGCCTTCAACCGCCACGGCTGGGCACCAGGACCC
AAAGTGGTGACTGGTTATTCTGGGGAAGACTTGCCCTTGGTGGCTCCCGATAACGTATCTGCGGAG
GTGTTGAACTCTACACTGCTGAAGGTCAGCTGGTCACCAGTGCCCCAAACCACCCTGAGGGGCCAT
CTTGGAGGATACACAGTGAACTACTGGCAAACTCAGAGTCTGTTGACCTCTAAGAAAATTCCCTCA
GAGAGACAGTCTCTCACCATCCCTGGAAACAGGAGTCACACCATGGTGCCAGGACTGAAACCATTC
TCTGAATATAGTCTGACTGTCAACGTCTTTAACCGGAGAGGGAATGGGCCCCAAGCAGCAGTCCCA
TCTCTTTCACAACTCCACAAGGGAGTTCCTGAACAACCTCATAACCTGAAAGCCACAAACTTTCAGA
AAGACGCCATCACACTGGTGTGGGCGCCTCCGCACGAAACCAACGGCCATCTCACAGGATACCTTC
TGCAATACCAAACAGTTGTAGATAATGAGACTCTGGGTGAGCTGAACTCTGTGAACATCTCTGGAG
CAGACACGACACAGTGGGTTTTGCGTGATCTGCAGGAAGACAACTCATATAAGTTCTACCTGAGTG
CGTGCACGCAGGTGGGCTGTGGGTCAGCCATCAGTGAGAAAGGAGGCACCGACCCTGCAGCATTC
GTGCTCAACGTATCGACCTTTGTAAGAGACACTTCTGCAAGAATCAGCTGGAGAACCAGAGGAGA
GCATTCAGATTCACAGCTCTATGTTGCTATAATGAACCACCGTGATGGTATCTGGCAGCTTTCAGAGG
CTTTAAATACATCTAAAGGTTTCCATCTGGTTGAAGGACTCAGCCCAGGAACTGTGTACACAGTCCG
GCTGCAGGCTTCACGCAGGGTTGGTGTCCCTAGCATTTTTGAAGAGGTTTTCAAGACCAGGAAAG
CTTCTAGCAGACAAGAGATGGGATCTCAGGAGTGGTTTGTGGGGATGATGTGCGCGGTGGCTTTG
CTAACTCTGCTTGTCCTCATCGGCTGTTTTGTGCTCAAGAACAAAGGTGGCAAATACGCAGTAAAGG
AAAAGGAGAACATTCCAGCTGATCCTGAATCTCATGGCATGGATGAAGAAACACCCTGTGAATACA
GTGACAATGATGAGAAGCCGCTGAAGAGCAGCCAGCAATTGTTCCGAGACGCCACAGAGGATGAT
CGCAGTGAGGATAGCTCTGAGATATCAGAGGATGATGACTGCGAGTTCAATGAAGATGGGTCATTC
ATTGGTGAATACTCTGGGTACATGCGCAGGACGTCCACGGCGGTCAACGGATTGAATGGACATACC
CCAGGAACTTCTTAACAGTGGCCCAATTTTATGAACTTTTTAAACAATTCAACAGTAAGAAAGGTTTT
GTTTGAATGGTGTGCCATGAATGTGCCTATGCTTCTGCGTGATTGGGCCAGTTTTTCTACAGTATTAATA
TGAAGGTTGAAAATTTATGATTCTGCGCAACACAAAACAACTGTTTCAAAACTTTGAGTTTTTTAAGC
AAAACTTTTATGCTACTTTTTCCATTCAATTAAAAAAAA

Isoform 2 cDNA sequece

ATTCTGCTCATCTCTTCTGACAGGAGCTTAACGCCGACTTAAATCAGATCTTTCTCTTGAAGGAGTCGG
TAAGAAGCTCCTGACGAGTGTTTTGAGCATATCCTTCATCTTTCTCTGGCTGGGATGAGGGGGCTCAG
GAGTCTCAGTGTTGGGCTCCTGTCGGCCTTCTTCTCCACCACCTTTGGTCTTTATTTCCCTCCTCACGT
GGAGCAGCTGCCCACCATCACCGCTCAGACCTCAGGCACTATCATCGCCCTACCATATGATGAGCGAT
ACATATTCAAGTGTGAAGCCAAAGGAAACCCAAGACCTACGTTCCGCTGGACAAAAGATGGGATG

GATTTTGACCCACATAAAGACCCTCAGGTCACTACAGAAGAAAATACTGGAACCTTTGAGATACCA
AATAAAAAAGATAAAGTGAAATACCAGGGAAAGTACAGATGTTACGCCTCCAACAAACTCGGAAC
TGCAACTTCAGAAGAAGCCGAATTTATTGTGCCTATGGTACCCAAGTTTCCCAAGGAGAAGATAGA
GCCAATTGTGGTCACAGAGGGTGATTCGGTGGTTTTGGAGTGTAACCCGCCACAAGGGATTCCACC
CTGGCAGCTGTACTGGATGACCAATGATCTCCGGCACATTGAGCAGAATGAGAGGGTGTCCATGGG
CCTCAGTGGGAATCTGTACTTCTCTAATACGCTGGTGTCAGACAGTCATGATGACTACTGCTGCTTCG
CCTCCTTCTTCAAAATACGCACAATCGTCCAGAAACCCCGCATGGTCCTCAAGGTCAAGCCAGTTGA
TGCAAAGCAAGAGGATGGTGATGCCAAGATTGTTCAGGAGAGGAAGCCAAGCATCTTGGTTCCCT
CCAGTCATTCAACGACTTTCCTGAAAAAAGGAGAAGAGCTGGAGCTGGAGTGTATTGCAGAAGGA
CTGCCTACACCGGAGGTAGAATGGATCAAGATATGGGGTGACTTACCAAAGCGCACGCAGATAAAG
AACTTCGGAAAGCTTCTGACAATACCAAACATCAGAGAAGAGGACGAAGGAAAATACATGTGTAA
AGCCAAGAATCAGCATGGTGAAGATGTGCATCATTTCCAGGTCGAAGTAGAGGAGCCACCGAGCT
GGCAGAAGGAGCCGCTGAAGAGTCAGTTAGCATGGATTGGATCTGACATCCACATCAACTGTTCTG
CTACCGGAAAACCGCAACCCACAATCACCTGGAAAAGAAACGGCAAGCCCCTCGATGATTTCCCTT
CCACCCATCACAAAATGCTTGACGACAGAATAATCATACTCAAAGCGGAGCAGAAAGATACCGCTG
TGTACCAGTGTGAGGCCTCCAACAAACACGGCAGCCTACTGGCCAATGCTAATGTGCTAGTCATGAA
TCACCCTCCTTTGATTCTGACGAGGAATTATCTGGAGTATGCAACCATGTTGGGCAAGAGTGTGATT
ATGGACTGCAATGTCTTTAGTTCACCACCTGCCACCCTTAACTGGAGAAGAGAAGACCCAGAGGGC
TCTGTAGATGGAGAGAGATATTCTCTGCTGAAGAATGGCTCCCTGCAGATTCACAAAGTCGAGATG
GAAGATATGGGCCAGTACAAATGCTTGGCAAACAACTCCGAGGGCTCTGCAAGCATCACCACTGAA
CTTTTCATTAAAGATTCCACTAGGATCGTGGAGGCTCCTCAAGACATGAAGGTCATCCGGGGATCTG
TGGCCGAGCTGGAGTGCCAGGTGGAGTGCGACCCGTCTCTGAGAAGAGAGCTGGAGTTCGTTTG
GCTCAAAGATGGGATAAATATCCTCTCGAACTCCTCAGAAAGGTATTTTACAGATGAAGGAATTCTT
CAGATATTCAACGTGAGTCACAGTGATCAAGGGAATTACACCTGTATTGTTCGAACATCCCTGGACC
AAGATACAGCTTCTGCATACATAACAGTCTTAGATGTCCCCGATCCTCCGGTTGAATTATTGCTCAGT
GATCTGAAAGACCGCAGTGTGAAGCTCCACTGGGTGAACACCAGTGATCACAACAGTCCCATAACA
GAGTTCATCATCCAGTACGAAGAGAGCCACTGGGAGCCAGAGAAGTGGAAAGATCTTCTTCGAGT
GTCTAGCAGGAAGTTCTCAGCCCCCTTGGCTCTGTACTGCCACATTAACTACCAGTTCAGAGTCATCG
CTGTCAATGAAATAGGGAGAAGTCATCCCAGTATGCCCTCAGGAAGATATAAAACACCTCCATGTGC
TCCTGACAGGAACCCAGAAAACATTAAGATTGAGGGTCACCTGCCACATCAGATGGACATCAGCTG
GGAGCCGCTCTTGCCGGTGGAGCACAACGGTCCCGGTCTGGAATACAAACTGAGCTACAGGCTCCT
GGGTGTCGAGGACAGTTGGAAAGAGCAGATGGTAAAGAGACACTCTTTTGTGGTGCGAGACACC
CCGACATTCGTCCCCTATGAAGTCAAAATCCAGGCCTTCAACCGCCACGGCTGGGCACCAGGACCC
AAAGTGGTGACTGGTTATTCTGGGGAAGACTTGCCCTTGGTGGCTCCCGATAACGTATCTGCGGAG
GTGTTGAACTCTACACTGCTGAAGGTCAGCTGGTCACCAGTGCCCCAAACCACCCTGAGGGGCCAT
CTTGGAGGATACACAGTGAACTACTGGCAAACTCAGAGTCTGTTGACCTCTAAGAAAATTCCCTCA
GAGAGACAGTCTCTCACCATCCCTGGAAACAGGAGTCACACCATGGTGCCAGGACTGAAACCATTC
TCTGAATATAGTCTGACTGTCAACGTCTTTAACCGGAGAGGGAATGGGCCCCAAGCAGCAGTCCCA
TCTCTTTCACAACTCCACAAGGGAGTTCCTGAACAACCTCATAACCTGAAAGCCACAAACTTTCAGA
AAGACGCCATCACACTGGTGTGGGCGCCTCCGCACGAAACCAACGGCCATCTCACAGGATACCTTC
TGCAATACCAAACAGTTGTAGATAATGAGACTCTGGGTGAGCTGAACTCTGTGAACATCTCTGGAG
CAGACACGACACAGTGGGTTTTGCGTGATCTGCAGGAAGACAACTCATATAAGTTCTACCTGAGTG
CGTGCACGCAGGTGGGCTGTGGGTCAGCCATCAGTGAGAAAGGAGGCACCGACCCTGCAGCATGT
GATGGTATCTGGCAGCTTTCAGAGGCTTTAAATACATCTAAAGGTTTCCATCTGGTTGAAGGACTCA

GCCCAGGAACTGTGTACACAGTCCGGCTGCAGGCTTCACGCAGGGTTGGTGTCCCTAGCATTTTTG
AAGAGGTTTTCAAGACCAGGAAAGCTTCTAGCAGACAAGAGATGGGATCTCAGGAGTGGTTTGTG
GGGATGATGTGCGCGGTGGCTTTGCTAACTCTGCTTGTCCTCATCGGCTGTTTTGTGCTCAAGAACA
AAGGTGGCAAATACGCAGTAAAGGAAAAGGAGAACATTCCAGCTGATCCTGAATCTCATGGCATG
GATGAAGAAACACCCTGTGAATACAGTGACAATGATGAGAAGCCGCTGAAGAGCAGCCAGCAATT
GTTCCGAGACGCCACAGAGGATGATCGCAGTGAGGATAGCTCTGAGATATCAGAGGATGATGACTG
CGAGTTCAATGAAGATGGGTCATTCATTGGTGAATACTCTGGGTACATGCGCAGGACGTCCACGGC
GGTCAACGGATTGAATGGACATACCCCAGGAACTTCTTAACAGTGGCCCAATTTTATGAACTTTTTAA
ACAATTCAACAGTAAGAAAGGTTTTGTTTGAATGGTGTGCCATGAATGTGCCTATGCTTCTGCGTGATT
GGGCCAGTTTTTCTACAGTATTAATATGAAGGTTGAAAATTTATGATTCTGCGCAACACAA

Isoform 3 cDNA sequence

ATTCTGCTCATCTCTTCTGACAGGAGCTTAACGCCGACTTAAATCAGATCTTTCTCTTGAAGGAGTCGG
TAAGAAGCTCCTGACGAGTGTTTTGAGCATATCCTTCATCTTTCTCTGGCTGGGATGAGGGGGCTCAG
GAGTCTCAGTGTTGGGCTCCTGTCGGCCTTCTTCTCCACCACCTTTGGTCTTTATTTCCCTCCTCACGT
GGAGCAGCTGCCCACCATCACCGCTCAGACCTCAGGCACTATCATCGCCCTACCATATGATGAGCGAT
ACATATTCAAGTGTGAAGCCAAAGGAAACCCAAGACCTACGTTCCGCTGGACAAAAGATGGGATG
GATTTTGACCCACATAAAGACCCTCAGGTCACTACAGAAGAAAATACTGGAACCTTTGAGATACCA
AATAAAAAAGATAAAGTGAAATACCAGGGAAAGTACAGATGTTACGCCTCCAACAAACTCGGAAC
TGCAACTTCAGAAGAAGCCGAATTTATTGTGCCTATGGTACCCAAGTTTCCCAAGGAGAAGATAGA
GCCAATTGTGGTCACAGAGGGTGATTCGGTGGTTTTGGAGTGTAACCCGCCACAAGGGATTCCACC
CTGGCAGCTGTACTGGATGACCAATGATCTCCGGCACATTGAGCAGAATGAGAGGGTGTCCATGGG
CCTCAGTGGGAATCTGTACTTCTCTAATACGCTGGTGTCAGACAGTCATGATGACTACTGCTGCTTCG
CCTCCTTCTTCAAAATACGCACAATCGTCCAGAAACCCCGCATGGTCCTCAAGGTCAAGCCAGTTGA
TGCAAAGCAAGAGGATGGTGATGCCAAGATTGTTCAGGAGAGGAAGCCAAGCATCTTGGTTCCCT
CCAGTCATTCAACGACTTTCCTGAAAAAAGGAGAAGAGCTGGAGCTGGAGTGTATTGCAGAAGGA
CTGCCTACACCGGAGGTAGAATGGATCAAGATATGGGGTGACTTACCAAAGCGCACGCAGATAAAG
AACTTCGGAAAGCTTCTGACAATACCAAACATCAGAGAAGAGGACGAAGGAAAATACATGTGTAA
AGCCAAGAATCAGCATGGTGAAGATGTGCATCATTTCCAGGTCGAAGTAGAGGAGCCACCGAGCT
GGCAGAAGGAGCCGCTGAAGAGTCAGTTAGCATGGATTGGATCTGACATCCACATCAACTGTTCTG
CTACCGGAAAACCGCAACCCACAATCACCTGGAAAAGAAACGGCAAGCCCCTCGATGATTTCCCTT
CCACCCATCACAAAATGCTTGACGACAGAATAATCATACTCAAAGCGGAGCAGAAAGATACCGCTG
TGTACCAGTGTGAGGCCTCCAACAAACACGGCAGCCTACTGGCCAATGCTAATGTGCTAGTCATGAA
TCACCCTCCTTTGATTCTGACGAGGAATTATCTGGAGTATGCAACCATGTTGGGCAAGAGTGTGATT
ATGGACTGCAATGTCTTTAGTTCACCACCTGCCACCCTTAACTGGAGAAGAGAAGACCCAGAGGGC
TCTGTAGATGGAGAGAGATATTCTCTGCTGAAGAATGGCTCCCTGCAGATTCACAAAGTCGAGATG
GAAGATATGGGCCAGTACAAATGCTTGGCAAACAACTCCGAGGGCTCTGCAAGCATCACCACTGAA
CTTTTCATTAAAGATTCCACTAGGATCGTGGAGGCTCCTCAAGACATGAAGGTCATCCGGGGATCTG
TGGCCGAGCTGGAGTGCCAGGTGGAGTGCGACCCGTCTCTGAGAAGAGAGCTGGAGTTCGTTTG
GCTCAAAGATGGGATAAATATCCTCTCGAACTCCTCAGAAAGGTATTTTACAGATGAAGGAATTCTT
CAGATATTCAACGTGAGTCACAGTGATCAAGGGAATTACACCTGTATTGTTCGAACATCCCTGGACC
AAGATACAGCTTCTGCATACATAACAGTCTTAGATGTCCCCGATCCTCCGGTTGAATTATTGCTCAGT
GATCTGAAAGACCGCAGTGTGAAGCTCCACTGGGTGAACACCAGTGATCACAACAGTCCCATAACA
GAGTTCATCATCCAGTACGAAGAGAGCCACTGGGAGCCAGAGAAGTGGAAAGATCTTCTTCGAGT

**GTCTAGCAGGAAGTTCTCAGCCCCCTTGGCTCTGTACTGCCACATTAACTACCAGTTCAGAGTCATCG
CTGTCAATGAAATAGGGAGAAGTCATCCCAGTATGCCCTCAGGAAGATATAAAACACCTCCATGTGC
TCCTGACAGGAACCCAGAAAACATTAAGATTGAGGGTCACCTGCCACATCAGATGGACATCAGCTG
GGAGCCGCTCTTGCCGGTGGAGCACAACGGTCCCGGTCTGGAATACAAACTGAGCTACAGGCTCCT
GGGTGTCGAGGACAGTTGGAAAGAGCAGATGGTAAAGAGACACTCTTTTGTGGTGCGAGACACC
CCGACATTCGTCCCCTATGAAGTCAAAATCCAGGCCTTCAACCGCCACGGCTGGGCACCAGGACCC
AAAGTGGTGACTGGTTATTCTGGGGAAGACTTGCCCTTGGTGGCTCCCGATAACGTATCTGCGGAG
GTGTTGAACTCTACACTGCTGAAGGTCAGCTGGTCACCAGTGCCCCAAACCACCCTGAGGGGCCAT
CTTGGAGGATACACAGTGAACTACTGGCAAACTCAGAGTCTGTTGACCTCTAAGAAAATTCCCTCA
GAGAGACAGTCTCTCACCATCCCTGGAAACAGGAGTCACACCATGGTGCCAGGACTGAAACCATTC
TCTGAATATAGTCTGACTGTCAACGTCTTTAACCGGAGAGGGAATGGGCCCCAAGCAGCAGTCCCA
TCTCTTTCACAACTCCACAAGGGAGTTCCTGAACAACCTCATAACCTGAAAGCCACAAACTTTCAGA
AAGACGCCATCACACTGGTGTGGGCGCCTCCGCACGAAACCAACGGCCATCTCACAGGATACCTTC
TGCAATACCAAACAGTTGTAGATAATGAGACTCTGGGTGAGCTGAACTCTGTGAACATCTCTGGAG
CAGACACGACACAGTGGGTTTTGCGTGATCTGCAGGAAGACAACTCATATAAGTTCTACCTGAGTG
CGTGCACGCAGGTGGGCTGTGGGTCAGCCATCAGTGAGAAAGGAGGCACCGACCCTGCAGCATTC
GTGCTCAACGTATCGACCTTTGTAAGAGACACTTCTGCAAGAATCAGCTGGAGAACCAGAGGAGA
GCATTCAGATTCACAGCTCTATGTTGCTATAATGAACCACCCTTCTAGCAGACAAGAGATGGGATCTC
AGGAGTGGTTTGTGGGGATGATGTGCGCGGTGGCTTTGCTAACTCTGCTTGTCCTCATCGGCTGTTT
TGTGCTCAAGAACAAAGGTGGCAAATACGCAGTAAAGGAAAAGGAGAACATTCCAGCTGATCCTG
AATCTCATGGCATGGATGAAGAAACACCCTGTGAATACAGTGACAATGATGAGAAGCCGCTGAAGA
GCAGCCAGCAATTGTTCCGAGACGCCACAGAGGATGATCGCAGTGAGGATAGCTCTGAGATATCAG
AGGATGATGACTGCGAGTTCAATGAAGATGGGTCATTCATTGGTGAATACTCTGGGTACATGCGCA
GGACGTCCACGGCGGTCAACGGATTGAATGGACATACCCCAGGAACTTCTTAA**CAGTGGCCCAATT
TTATGAACTTTTTAAACAATTCAACAGTAAGAAAGGTTTTGTTTGAATGGTGTGCCATGAATGTGCCTA
TGCTTCTGCGTGATTGGGCCAGTTTTTCTACAGTATTAATATGAAGGTTGAAAATTTATGATTCTGCGC

Isoform 4 cDNA sequece

ATTCTGCTCATCTCTTCTGACAGGAGCTTAACGCCGACTTAAATCAGATCTTTCTCTTGAAGGAGTCGG
TAAGAAGCTCCTGACGAGTGTTTTGAGCATATCCTTCATCTTTCTCTGGCTGGG**ATGAGGGGGCTCAG
GAGTCTCAGTGTTGGGCTCCTGTCGGCCTTCTTCTCCACCACCTTTGGTCTTTATTTCCCTCCTCACGT
GGAGCAGCTGCCCACCATCACCGCTCAGACCTCAGGCACTATCATCGCCCTACCATATGATGAGCGAT
ACATATTCAAGTGTGAAGCCAAAGGAAACCCAAGACCTACGTTCCGCTGGACAAAAGATGGGATG
GATTTTGACCCACATAAAGACCCTCAGGTCACTACAGAAGAAAATACTGGAACCTTTGAGATACCA
AATAAAAAAGATAAAGTGAAATACCAGGGAAAGTACAGATGTTACGCCTCCAACAAACTCGGAAC
TGCAACTTCAGAAGAAGCCGAATTTATTGTGCCTATGGTACCCAAGTTTCCCAAGGAGAAGATAGA
GCCAATTGTGGTCACAGAGGGTGATTCGGTGGTTTTGGAGTGTAACCCGCCACAAGGGATTCCACC
CTGGCAGCTGTACTGGATGACCAATGATCTCCGGCACATTGAGCAGAATGAGAGGGTGTCCATGGG
CCTCAGTGGGAATCTGTACTTCTCTAATACGCTGGTGTCAGACAGTCATGATGACTACTGCTGCTTCG
CCTCCTTCTTCAAAATACGCACAATCGTCCAGAAACCCCGCATGGTCCTCAAGGTCAAGCCAGTTGA
TGCAAAGCAAGAGGATGGTGATGCCAAGATTGTTCAGGAGAGGAAGCCAAGCATCTTGGTTCCCT
CCAGTCATTCAACGACTTTCCTGAAAAAAGGAGAAGAGCTGGAGCTGGAGTGTATTGCAGAAGGA
CTGCCTACACCGGAGGTAGAATGGATCAAGATATGGGGTGACTTACCAAAGCGCACGCAGATAAAG
AACTTCGGAAAGCTTCTGACAATACCAAACATCAGAGAAGAGGACGAAGGAAAATACATGTGTAA**

**AGCCAAGAATCAGCATGGTGAAGATGTGCATCATTTCCAGGTCGAAGTAGAGGAGCCACCGAGCT
GGCAGAAGGAGCCGCTGAAGAGTCAGTTAGCATGGATTGGATCTGACATCCACATCAACTGTTCTG
CTACCGGAAAACCGCAACCCACAATCACCTGGAAAAGAAACGGCAAGCCCCTCGATGATTTCCCTT
CCACCCATCACAAAATGCTTGACGACAGAATAATCATACTCAAAGCGGAGCAGAAAGATACCGCTG
TGTACCAGTGTGAGGCCTCCAACAAACACGGCAGCCTACTGGCCAATGCTAATGTGCTAGTCATGAA
TCACCCTCCTTTGATTCTGACGAGGAATTATCTGGAGTATGCAACCATGTTGGGCAAGAGTGTGATT
ATGGACTGCAATGTCTTTAGTTCACCACCTGCCACCCTTAACTGGAGAAGAGAAGACCCAGAGGGC
TCTGTAGATGGAGAGAGATATTCTCTGCTGAAGAATGGCTCCCTGCAGATTCACAAAGTCGAGATG
GAAGATATGGGCCAGTACAAATGCTTGGCAAACAACTCCGAGGGCTCTGCAAGCATCACCACTGAA
CTTTTCATTAAAGATTCCACTAGGATCGTGGAGGCTCCTCAAGACATGAAGGTCATCCGGGGATCTG
TGGCCGAGCTGGAGTGCCAGGTGGAGTGCGACCCGTCTCTGAGAAGAGAGCTGGAGTTCGTTTG
GCTCAAAGATGGGATAAATATCCTCTCGAACTCCTCAGAAAGGTATTTTACAGATGAAGGAATTCTT
CAGATATTCAACGTGAGTCACAGTGATCAAGGGAATTACACCTGTATTGTTCGAACATCCCTGGACC
AAGATACAGCTTCTGCATACATAACAGTCTTAGATGTCCCCGATCCTCCGGTTGAATTATTGCTCAGT
GATCTGAAAGACCGCAGTGTGAAGCTCCACTGGGTGAACACCAGTGATCACAACAGTCCCATAACA
GAGTTCATCATCCAGTACGAAGAGAGCCACTGGGAGCCAGAGAAGTGGAAAGATCTTCTTCGAGT
GTCTAGCAGGAAGTTCTCAGCCCCCTTGGCTCTGTACTGCCACATTAACTACCAGTTCAGAGTCATCG
CTGTCAATGAAATAGGGAGAAGTCATCCCAGTATGCCCTCAGGAAGATATAAAACACCTCCATGTGC
TCCTGACAGGAACCCAGAAAACATTAAGATTGAGGGTCACCTGCCACATCAGATGGACATCAGCTG
GGAGCCGCTCTTGCCGGTGGAGCACAACGGTCCCGGTCTGGAATACAAACTGAGCTACAGGCTCCT
GGGTGTCGAGGACAGTTGGAAAGAGCAGATGGTAAAGAGACACTCTTTTGTGGTGCGAGACACC
CCGACATTCGTCCCCTATGAAGTCAAAATCCAGGCCTTCAACCGCCACGGCTGGGCACCAGGACCC
AAAGTGGTGACTGGTTATTCTGGGGAAGACTTGCCCTTGGTGGCTCCCGATAACGTATCTGCGGAG
GTGTTGAACTCTACACTGCTGAAGGTCAGCTGGTCACCAGTGCCCCAAACCACCCTGAGGGGCCAT
CTTGGAGGATACACAGTGAACTACTGGCAAACTCAGAGTCTGTTGACCTCTAAGAAAATTCCCTCA
GAGAGACAGTCTCTCACCATCCCTGGAAACAGGAGTCACACCATGGTGCCAGGACTGAAACCATTC
TCTGAATATAGTCTGACTGTCAACGTCTTTAACCGGAGAGGGAATGGGCCCCAAGCAGCAGTCCCA
TCTCTTTCACAACTCCACAAGGGAGTTCCTGAACAACCTCATAACCTGAAAGCCACAAACTTTCAGA
AAGACGCCATCACACTGGTGTGGGCGCCTCCGCACGAAACCAACGGCCATCTCACAGGATACCTTC
TGCAATACCAAACAGTTGTAGATAATGAGACTCTGGGTGAGCTGAACTCTGTGAACATCTCTGGAG
CAGACACGACACAGTGGGTTTTGCGTGATCTGCAGGAAGACAACTCATATAAGTTCTACCTGAGTG
CGTGCACGCAGGTGGGCTGTGGGTCAGCCATCAGTGAGAAAGGAGGCACCGACCCTGCAGCATCT
TCTAGCAGACAAGAGATGGGATCTCAGGAGTGGTTTGTGGGGATGATGTGCGCGGTGGCTTTGCT
AACTCTGCTTGTCCTCATCGGCTGTTTTGTGCTCAAGAACAAAGGTGGCAAATACGCAGTAAAGGA
AAAGGAGAACATTCCAGCTGATCCTGAATCTCATGGCATGGATGAAGAAACACCCTGTGAATACAG
TGACAATGATGAGAAGCCGCTGAAGAGCAGCCAGCAATTGTTCCGAGACGCCACAGAGGATGATC
GCAGTGAGGATAGCTCTGAGATATCAGAGGATGATGACTGCGAGTTCAATGAAGATGGGTCATTCA
TTGGTGAATACTCTGGGTACATGCGCAGGACGTCCACGGCGGTCAACGGATTGAATGGACATACCC
CAGGAACTTCTTAA**CAGTGGCCCAATTTTATGAACTTTTTAAACAATTCAACAGTAAGAAAGGTTTTG
TTTGAATGGTGTGCCATGAATGTGCCTATGCTTCTGCGTGATTGGGCCAGTTTTTCTACAGTATTAATAT
GAAGGTTGAAAATTTATGATTCTGCGCAA

Notes: The sequence and diagram are color-to-color matching

The protein sequence of camel isoforms

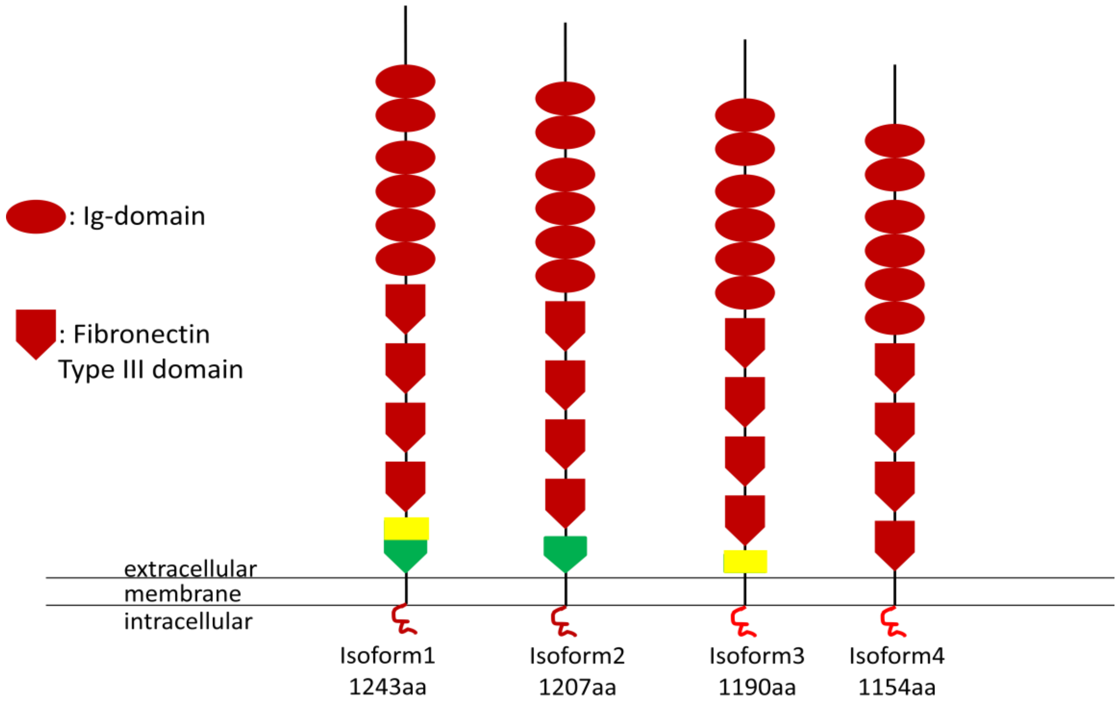

Isoform 1 protein sequence 1243aa

MRGLRSLSVGLLSAFFSTTFGLYFPPHVEQLPTITAQTSGTIIALPYDERYIFKCEAKGNPRPTFRWTKDGM
DFDPHKDPQVTTEENTGTFEIPNKKDKVKYQGKYRCYASNKLGTATSEEAEFIVPMVPKFPKEKIEPIVVTE
GDSVVLECNPPQGIPPWQLYWMTNDLRHIEQNERVSMGLSGNLYFSNTLVSDSHDDYCCFASFFKIRTI
VQKPRMVLKVKPVDAKQEDGDAKIVQERKPSILVPSSHSTTFLKKGEELELECIAEGLPTPEVEWIKIWGD
LPKRTQIKNFGKLLTIPNIREEDEGKYMCKAKNQHGEDVHHFQVEVEEPPSWQKEPLKSQLAWIGSDIHI
NCSATGKPQPTITWKRNGKPLDDFPSTHHKMLDDRIIILKAEQKDTAVYQCEASNKHGSLLANANVLVM
NHPPLILTRNYLEYATMLGKSVIMDCNVFSSPPATLNWRREDPEGSVDGERYSLLKNGSLQIHKVEMED
MGQYKCLANNSEGSASITTELFIKDSTRIVEAPQDMKVIRGSVAELECQVECDPSLRRELEFVWLKDGINI
LSNSSERYFTDEGILQIFNVSHSDQGNYTCIVRTSLDQDTASAYITVLDVPDPPVELLLSDLKDRSVKLHWV
NTSDHNSPITEFIIQYEESHWEPEKWKDLLRVSSRKFSAPLALYCHINYQFRVIAVNEIGRSHPSMPSGRYK
TPPCAPDRNPENIKIEGHLPHQMDISWEPLLPVEHNGPGLEYKLSYRLLGVEDSWKEQMVKRHSFVVR
DTPTFVPYEVKIQAFNRHGWAPGPKVVTGYSGEDLPLVAPDNVSAEVLNSTLLKVSWSPVPQTTLRGHL
GGYTVNYWQTQSLLTSKKIPSERQSLTIPGNRSHTMVPGLKPFSEYSLTVNVFNRRGNGPQAAVPSLSQL
HKGVPEQPHNLKATNFQKDAITLVWAPPHETNGHLTGYLLQYQTVVDNETLGELNSVNISGADTTQWV
LRDLQEDNSYKFYLSACTQVGCGSAISEKGGTDPAAFVLNVSTFVRDTSARISWRTRGEHSDSQLYVAIM
NHRDGIWQLSEALNTSKGFHLVEGLSPGTVYTVRLQASRRVGVPSIFEEVFKTRKASSRQEMGSQEWFV
GMMCAVALLTLLVLIGCFVLKNKGGKYAVKEKENIPADPESHGMDEETPCEYSDNDEKPLKSSQQLFRDA
TEDDRSEDSSEISEDDDCEFNEDGSFIGEYSGYMRRTSTAVNGLNGHTPGTS

Isoform 2 -proteins sequence 1207aa

MRGLRSLSVGLLSAFFSTTFGLYFPPHVEQLPTITAQTSGTIIALPYDERYIFKCEAKGNPRPTFRWTKDGM
DFDPHKDPQVTTEENTGTFEIPNKKDKVKYQGKYRCYASNKLGTATSEEAEFIVPMVPKFPKEKIEPIVVTE
GDSVVLECNPPQGIPPWQLYWMTNDLRHIEQNERVSMGLSGNLYFSNTLVSDSHDDYCCFASFFKIRTI
VQKPRMVLKVKPVDAKQEDGDAKIVQERKPSILVPSSHSTTFLKKGEELELECIAEGLPTPEVEWIKIWGD
LPKRTQIKNFGKLLTIPNIREEDEGKYMCKAKNQHGEDVHHFQVEVEEPPSWQKEPLKSQLAWIGSDIHI
NCSATGKPQPTITWKRNGKPLDDFPSTHHKMLDDRIIILKAEQKDTAVYQCEASNKHGSLLANANVLVM
NHPPLILTRNYLEYATMLGKSVIMDCNVFSSPPATLNWRREDPEGSVDGERYSLLKNGSLQIHKVEMED
MGQYKCLANNSEGSASITTELFIKDSTRIVEAPQDMKVIRGSVAELECQVECDPSLRRELEFVWLKDGINI
LSNSSERYFTDEGILQIFNVSHSDQGNYTCIVRTSLDQDTASAYITVLDVPDPPVELLLSDLKDRSVKLHWV
NTSDHNSPITEFIIQYEESHWEPEKWKDLLRVSSRKFSAPLALYCHINYQFRVIAVNEIGRSHPSMPSGRYK
TPPCAPDRNPENIKIEGHLPHQMDISWEPLLPVEHNGPGLEYKLSYRLLGVEDSWKEQMVKRHSFVVR
DTPTFVPYEVKIQAFNRHGWAPGPKVVTGYSGEDLPLVAPDNVSAEVLNSTLLKVSWSPVPQTTLRGHL
GGYTVNYWQTQSLLTSKKIPSERQSLTIPGNRSHTMVPGLKPFSEYSLTVNVFNRRGNGPQAAVPSLSQL
HKGVPEQPHNLKATNFQKDAITLVWAPPHETNGHLTGYLLQYQTVVDNETLGELNSVNISGADTTQWV
LRDLQEDNSYKFYLSACTQVGCGSAISEKGGTDPAACDGIWQLSEALNTSKGFHLVEGLSPGTVYTVRLQ
ASRRVGVPSIFEEVFKTRKASSRQEMGSQEWFVGMMCAVALLTLLVLIGCFVLKNKGGKYAVKEKENIPA
DPESHGMDEETPCEYSDNDEKPLKSSQQLFRDATEDDRSEDSSEISEDDDCEFNEDGSFIGEYSGYMRRT
STAVNGLNGHTPGTS

Isoform 3 protein sequence 1190aa

MRGLRSLSVGLLSAFFSTTFGLYFPPHVEQLPTITAQTSGTIIALPYDERYIFKCEAKGNPRPTFRWTKDGM
DFDPHKDPQVTTEENTGTFEIPNKKDKVKYQGKYRCYASNKLGTATSEEAEFIVPMVPKFPKEKIEPIVVTE
GDSVVLECNPPQGIPPWQLYWMTNDLRHIEQNERVSMGLSGNLYFSNTLVSDSHDDYCCFASFFKIRTI
VQKPRMVLKVKPVDAKQEDGDAKIVQERKPSILVPSSHSTTFLKKGEELELECIAEGLPTPEVEWIKIWGD
LPKRTQIKNFGKLLTIPNIREEDEGKYMCKAKNQHGEDVHHFQVEVEEPPSWQKEPLKSQLAWIGSDIHI
NCSATGKPQPTITWKRNGKPLDDFPSTHHKMLDDRIIILKAEQKDTAVYQCEASNKHGSLLANANVLVM
NHPPLILTRNYLEYATMLGKSVIMDCNVFSSPPATLNWRREDPEGSVDGERYSLLKNGSLQIHKVEMED
MGQYKCLANNSEGSASITTELFIKDSTRIVEAPQDMKVIRGSVAELECQVECDPSLRRELEFVWLKDGINI
LSNSSERYFTDEGILQIFNVSHSDQGNYTCIVRTSLDQDTASAYITVLDVPDPPVELLLSDLKDRSVKLHWV
NTSDHNSPITEFIIQYEESHWEPEKWKDLLRVSSRKFSAPLALYCHINYQFRVIAVNEIGRSHPSMPSGRYK
TPPCAPDRNPENIKIEGHLPHQMDISWEPLLPVEHNGPGLEYKLSYRLLGVEDSWKEQMVKRHSFVVR
DTPTFVPYEVKIQAFNRHGWAPGPKVVTGYSGEDLPLVAPDNVSAEVLNSTLLKVSWSPVPQTTLRGHL
GGYTVNYWQTQSLLTSKKIPSERQSLTIPGNRSHTMVPGLKPFSEYSLTVNVFNRRGNGPQAAVPSLSQL
HKGVPEQPHNLKATNFQKDAITLVWAPPHETNGHLTGYLLQYQTVVDNETLGELNSVNISGADTTQWV
LRDLQEDNSYKFYLSACTQVGCGSAISEKGGTDPAAFVLNVSTFVRDTSARISWRTRGEHSDSQLYVAIM
NHPSSRQEMGSQEWFVGMMCAVALLTLLVLIGCFVLKNKGGKYAVKEKENIPADPESHGMDEETPCEY
SDNDEKPLKSSQQLFRDATEDDRSEDSSEISEDDDCEFNEDGSFIGEYSGYMRRTSTAVNGLNGHTPGTS

Isoform 4 protein sequence 1154aa

MRGLRSLSVGLLSAFFSTTFGLYFPPHVEQLPTITAQTSGTIIALPYDERYIFKCEAKGNPRPTFRWTKDGM
DFDPHKDPQVTTEENTGTFEIPNKKDKVKYQGKYRCYASNKLGTATSEEAEFIVPMVPKFPKEKIEPIVVTE

GDSVVLECNPPQGIPPWQLYWMTNDLRHIEQNERVSMGLSGNLYFSNTLVSDSHDDYCCFASFFKIRTI
VQKPRMVLKVKPVDAKQEDGDAKIVQERKPSILVPSSHSTTFLKKGEELELECIAEGLPTPEVEWIKIWGD
LPKRTQIKNFGKLLTIPNIREEDEGKYMCKAKNQHGEDVHHFQVEVEEPPSWQKEPLKSQLAWIGSDIHI
NCSATGKPQPTITWKRNGKPLDDFPSTHHKMLDDRIIILKAEQKDTAVYQCEASNKHGSLLANANVLVM
NHPPLILTRNYLEYATMLGKSVIMDCNVFSSPPATLNWRREDPEGSVDGERYSLLKNGSLQIHKVEMED
MGQYKCLANNSEGSASITTELFIKDSTRIVEAPQDMKVIRGSVAELECQVECDPSLRRELEFVWLKDGINI
LSNSSERYFTDEGILQIFNVSHSDQGNYTCIVRTSLDQDTASAYITVLDVPDPPVELLLSDLKDRSVKLHWV
NTSDHNSPITEFIIQYEESHWEPEKWKDLLRVSSRKFSAPLALYCHINYQFRVIAVNEIGRSHPSMPSGRYK
TPPCAPDRNPENIKIEGHLPHQMDISWEPLLPVEHNGPGLEYKLSYRLLGVEDSWKEQMVKRHSFVVR
DTPTFVPYEVKIQAFNRHGWAPGPKVVTGYSGEDLPLVAPDNVSAEVLNSTLLKVSWSPVPQTTLRGHL
GGYTVNYWQTQSLLTSKKIPSERQSLTIPGNRSHTMVPGLKPFSEYSLTVNVFNRRGNGPQAAVPSLSQL
HKGVPEQPHNLKATNFQKDAITLVWAPPHETNGHLTGYLLQYQTVVDNETLGELNSVNISGADTTQWV
LRDLQEDNSYKFYLSACTQVGCGSAISEKGGTDPAASSSRQEMGSQEWFVGMMCAVALLTLLVLIGCFV
LKNKGGKYAVKEKENIPADPESHGMDEETPCEYSDNDEKPLKSSQQLFRDATEDDRSEDSSEISEDDDCE
FNEDGSFIGEYSGYMRRTSTAVNGLNGHTPGTS
